# Dual‐Responsive Hydrogels Engineer Anisotropic Cellular Microenvironment to Modulate Stem Cell Organization and Fate

**DOI:** 10.1002/smll.202600072

**Published:** 2026-05-14

**Authors:** Hongjuan Weng, Wen Chen, Lei He, Timo Rademakers, Sabine van Rijt, Monize C. Decarli, Katrien V. Bernaerts, Lorenzo Moroni

**Affiliations:** ^1^ Complex Tissue Regeneration Department MERLN Institute for Technology Inspired Regenerative Medicine Maastricht University Maastricht The Netherlands; ^2^ Sustainable Polymer Synthesis Group Aachen‐Maastricht Institute for Biobased Materials Maastricht University Maastricht The Netherlands; ^3^ Instructive Biomaterials Engineering Department MERLN Institute for Technology‐Inspired Regenerative Medicine Maastricht University Maastricht The Netherlands; ^4^ Department of Biomaterials and Biomedical Technology University Medical Center Groningen University of Groningen Groningen The Netherlands

**Keywords:** anisotropic hydrogels, biochemical cues, biomechanical cues, biophysical anisotropic cues, cell alignment, magnetic nanoparticles

## Abstract

Biophysical and biochemical cues in the local cellular microenvironment, including topography, matrix mechanics, and growth factors, significantly regulate stem cell fate. However, strategies for in vitro replicating such complex organized three‐dimensional (3D) cellular microenvironments and modulating cell alignment and differentiation by these cues in cell‐laden hydrogels are far less developed. This study introduces light‐responsive collagen peptide hydrogels mixed with magnetic nanoparticles as physical crosslinkers, leading to in situ formation of an organized network of human bone marrow mesenchymal stem cells (hMSCs) under magnetic‐driven anisotropy. Moreover, by simply tailoring the nanoparticle surface with dopamine methacrylamide, chemical nanoparticle crosslinkers with dual magnetic‐light‐responsiveness are developed to tune matrix mechanical dynamics without significantly changing hydrogel stiffness and components. The encapsulated hMSCs exhibit enhanced spreading, alignment, and differentiation into ligamentocytes/tenocytes in anisotropic hydrogels with faster mechanical dynamics and transforming growth factor beta‐3, contributing to the synergistic effects of these biophysical and biochemical cues. These simple manufacturing and conditioning strategies, which directly incorporate stimuli‐responsive nanoparticle crosslinkers into cell‐laden hydrogels, show great potential in developing advanced 3D organized in vitro models to modulate stem cell organization and fate.

## Graphical abstract

Collagen peptides hydrogels with tunable mechanical dynamics were developed by employing physical or chemical nanoparticle crosslinkers. Dual‐responsive hydrogels with magnetic‐driven alignment were fabricated under a low magnetic field and rapid UV exposure. The presence of biophysical anisotropy, enhanced biomechanical dynamics, and growth factors—as combined biophysical and biochemical cues—synergistically promoted the alignment and differentiation of encapsulated stem cells into ligamentocytes/tenocytes.

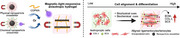

## 1 Introduction

Many human tissues are composed of organized cells surrounded by complex anisotropic extracellular matrix (ECM), such as ligaments, tendons, muscles, and nerves [1, 2]. Mimicking the aforementioned anisotropic ECM may pave the way for understanding cell‐ECM interactions in tissues and facilitate the rational design of constructs for tissue engineering. Hydrogels have been explored as synthetic ECM with 3D abundant aqueous networks, which could support cell proliferation in vitro [3]. However, fabricating anisotropic ECM‐mimicking matrix with an aligned 3D cell network by cell‐laden hydrogels remains challenging [4]. Biophysical signals (e.g., topography and mechanical cues) and biochemical factors (e.g., growth factors) presented in the cellular microenvironment play important roles in guiding cell behaviors, such as proliferation, migration, and differentiation, as well as tissue morphogenesis and homeostasis [5]. Yet, the synergistic effects of these signals are far less considered in regulating cell fate and alignment.

Various strategies have been studied to fabricate oriented structures [6, 7]. However, these strategies still need to be optimized when aiming at inducing and modulating 3D cell alignment and fate in situ to investigate cell‐material interactions and accelerate tissue regeneration. For example, 3D printing techniques (e.g., melt electrowritting and fused deposition modeling) and micropatterned chip‐based techniques can endow polymers with uniaxial morphology for guiding cell alignment [8, 9, 10]. Freeze‐casting techniques can fabricate aligned porous hydrogel scaffolds that lead cell growth in an aligned pattern [11]. Air drying with pre‐stretching approaches is developed to design hydrogels with a highly ordered hierarchical structure [12]. Despite these appealing approaches having demonstrated to influence cell activity and orientation according to initially designed criteria, they require cells to be seeded on the surface of scaffolds afterward, instead of developing a 3D anisotropic construct in situ.

Alternatively, cell‐laden hydrogels have also been synthesized to impart biophysical cues to cells, including cell alignment [7]. This can be more easily obtained with the use of external triggers, such as magnetic field, topographical guidance, and mechanical stimulation, which help controlling the activation of the designed biophysical stimuli [13]. Among such external triggers, magnetic field responsive iron oxide magnetic nanoparticles (MNPs) containing 3D anisotropic cell‐laden hydrogels have attracted increasing attentions [14]. For example, Demri et al. internalized MNPs into cells via endocytosis to obtain magnetic cells or spheroids for developing hydrogels with aligned cells or spheroids under a magnetic field [15]. Domingues et al. loaded MNPs into microfibers through electrospinning and cryo‐section techniques, and subsequently bioprinted anisotropic cell‐laden hydrogels [16]. Magnetospinning has been developed to fabricate aligned magnetic responsive nano‐ and microfibers, but these fibrous scaffolds require post cell seeding process rather than achieving 3D aligned cell network in situ [17, 18]. Braunmiller et al. created anisotropic hydrogels with magnetic rod‐shaped polyethylene glycol microgels that molded on a polydimethylsiloxame (PDMS) template [19]. Although these approaches successfully achieved 3D anisotropic cell networks in hydrogels, they typically require pre‐processing steps such as cell endocytosis MNPs, electrospinning of MNPs containing fibers, or PDMS molding of MNPs containing microgels. Therefore, it is significant to develop a more straightforward, one‐pot method for the induction of anisotropic 3D cell networks and ECMs by directly incorporating MNPs into cell‐laden hydrogels without any necessity of fiber fabrication or printing.

Cell behavior can also be influenced by hydrogel dynamics, including mechanical stiffness, viscoelasticity, and degradation kinetics [20]. Cells tend to spread in soft hydrogels but remain rounded in stiff hydrogels [21]. Hydrogels with faster relaxation rates facilitated cell‐ECM interactions, cell spreading, proliferation, and differentiation [22]. Moreover, biochemical factors, such as bioactive ligands and growth factors, can guide cell fate [23]. Bioactive ligands, such as arginine‐glycine‐aspartate (RGD), can promote mesenchymal stem cells (MSCs) adhesion and spreading in hydrogels [24]. Growth factors promote stem cell differentiation to target cell lines and induce them to produce ECM [25]. For example, transforming growth factor beta‐3 (TGF‐β3) can promote MSCs differentiating to ligamentocytes and remodeling matrix to mimic ECM in ligaments [26, 27]. However, the combined effect of the above biophysical topographic, biomechanical, and biochemical cues on regulating cell alignment and differentiation remains unclear.

**FIGURE 1:**
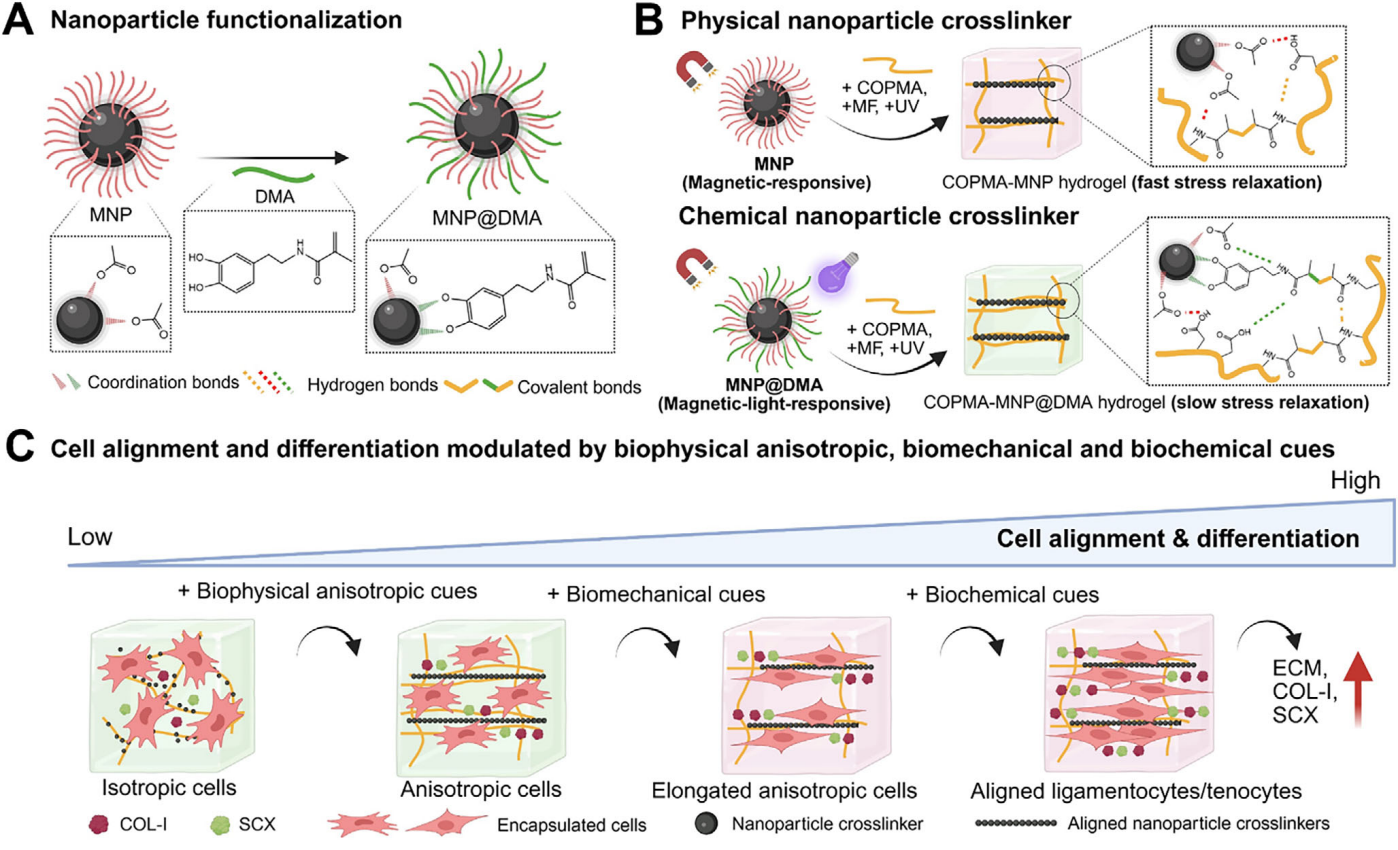
(A) Functionalization of magnetic nanoparticles (MNPs) by coating dopamine methacrylamide (DMA) on magnetic nanoparticles (MNP@DMA). (B) Physical and chemical nanoparticle crosslinkers in methacrylated collagen peptide (COPMA) hydrogels lead to different hydrogel mechanics. (C) Cell alignment and differentiation were modulated by a combination of biophysical anisotropic, biomechanical, and biochemical cues.

In this study, we developed MNP‐based 3D aligned constructs in a simple step and systematically modulated cell behaviors (e.g., alignment and phenotype) in the presence of topographical, biomechanical, and biochemical cues by employing a weak magnetic field (∼35 mT), hydrogel mechanical dynamics, and TGF‐β3, respectively (Figure 1). Anisotropic hydrogels were developed by simply mixing MNPs with methacrylated collagen peptides (COPMA) hydrogel precursors, followed by a simultaneous exposure of magnetic field (MF) and UV light for 3 min in one‐pot, owing to fast magnetic responsiveness. Isotropic COPMA hydrogels alone were used as a control. Furthermore, hydrogels with tunable mechanical dynamics were developed by introducing a dual magnetic‐light‐responsive nanoparticle crosslinker, namely MNPs coated by dopamine methacrylamide (MNP@DMA) via metal‐coordination bonding, referred to as COPMA‐MNP@DMA hydrogels (Figure 1A,B). The synergistic effects of biophysical anisotropic cues (e.g., without, short, or long magnetic field application times) and tunable biophysical mechanic cues (e.g., fast or slow stress‐relaxation) on cell alignment during matrix remodeling were investigated. Additionally, the dynamic cell‐laden hydrogels with different morphologies were cultured in the presence or absence of growth factors to further investigate the individual and combined effects of biophysical and biochemical cues on cell alignment and differentiation. (Figure 1C).

## 2 Results

### 2.1 Synthesis of MNP and MNP@DMA

**FIGURE 2:**
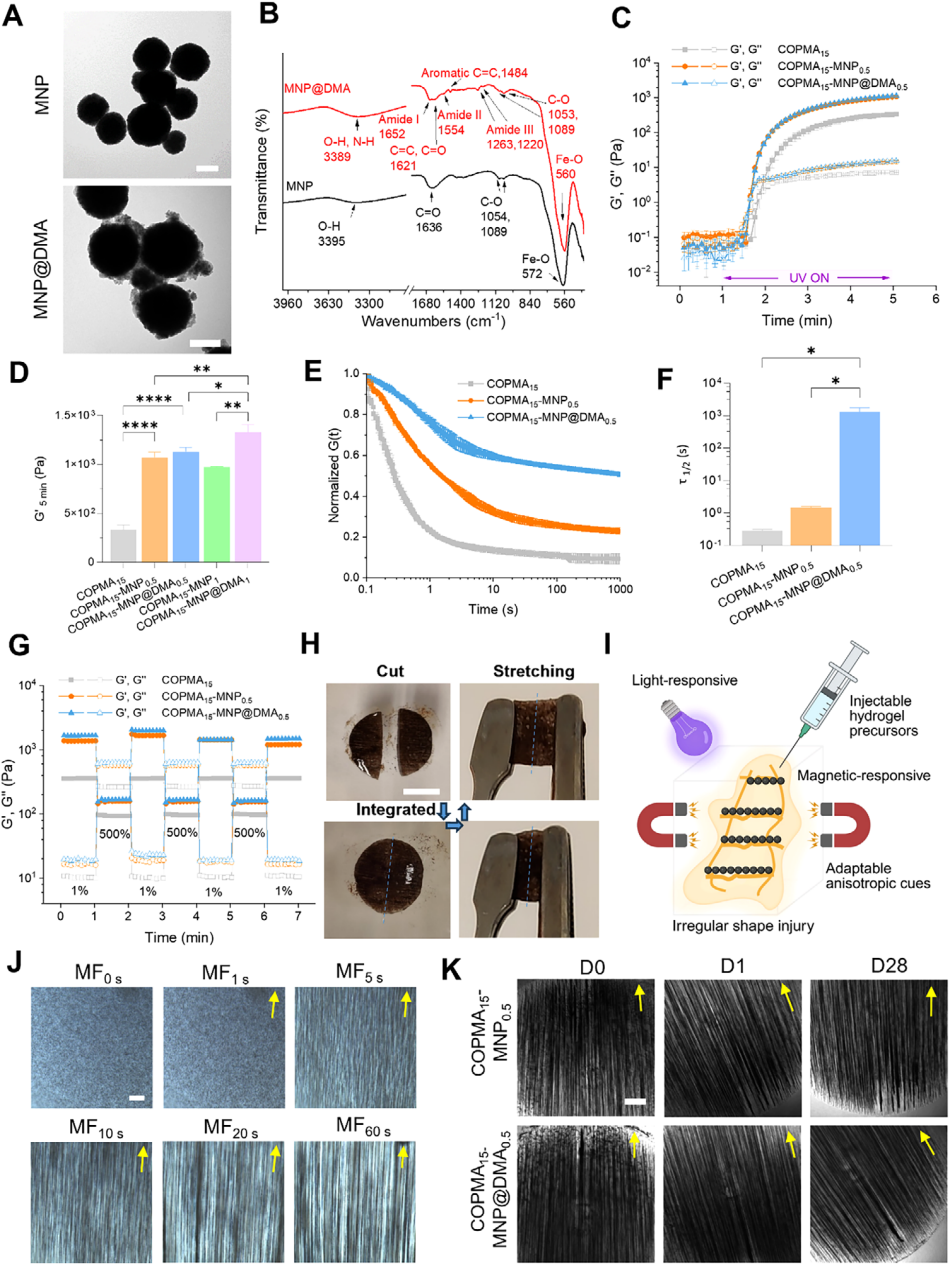
(A) TEM images of MNP and MNP@DMA, scale bar: 200 nm. (B) FTIR spectra of MNP and MNP@DMA. (C–H) Characterization of COPMA_15_, COPMA_15_‐MNP_0.5_, and COPMA_15_‐MNP@DMA_0.5_ hydrogels. C) Study of the hydrogel gelation process by using photorheology. (D) Storage modulus of hydrogels at 5 min (G′ _5 min_). (E) Stress‐relaxation of hydrogels under 20% strain. (F) Comparison of relaxation time of hydrogels. (G) Cyclic strain tests at 1% and 500% strains. Data were averaged from experiments repeated at least two times, and error bars represent standard deviation. (H) Self‐healing process of nanocomposite hydrogels, scale bar: 4 mm. (I) Scheme of nanocomposite hydrogels with magnetic‐light‐response properties and adaptable anisotropic cues. (J) Images of nanocomposite hydrogels with rapid magnetic‐response, scale bar: 1 mm. Yellow arrows indicate the direction of the magnetic field (MF). (K) Optical images of COPMA_15_‐MNP_0.5_ and COPMA_15_‐MNP@DMA_0.5_ hydrogels in medium on day 0, 1, and 28, scale bar: 500 µm.

MNPs have been widely used in tissue regeneration and cancer therapy because of their good biocompatibility, biodegradability, remote controllability, and full penetration to human body [28, 29, 30]. MNPs, which typically exhibit a high surface‐to‐volume ratio, could be coated with various functional materials to enhance their functionality and stability in an aqueous environment [31]. To form magnetic induced anisotropic structures, MNPs were synthesized with a diameter of 325 ± 54 nm (Figure 2A; Figure S1A). Furthermore, to strengthen the interactions between the hydrogels and nanoparticles, we functionalized the nanoparticle surface with the DMA molecule by metal‐coordination bonding (Figure 1A). DMA is a modified dopamine derivative with adhesion properties and radical crosslink properties [18]. Due to the metal‐coordination bonds and methacrylamide groups, MNP@DMA could act as dynamic nanoparticle crosslinkers with both magnetic‐ and light‐responsive properties, enabling a more precise control over the mechanical properties and structures of the hydrogels (Figure 1B). After functionalization, the diameter of MNP@DMA increased to 420 ± 52 nm, as measured via TEM (Figure 2A; Figure S1A). Correspondingly, the hydrodynamic diameter of MNP@DMA was also slightly higher than MNP (Figure S1B). MNP showed FTIR peaks at 3395 cm^−1^, 1636 cm^−1^, 1054, and 1089 cm^−1^, which corresponded to O─H, C═O, and C─O stretching, respectively (Figure 2B), indicating acetate groups formed coordination bonds with MNP, which could improve the hydrophilicity of MNP. The peak of Fe─O stretching was shifted from 572 cm^−1^ (MNP) to 560 cm^−1^ (MNP@DMA). In MNP@DMA spectrum, new peaks were shown at 1652, 1554, 1263, and 1220 cm^−1^, which were marked as amide I, II, and III peaks related to DMA, respectively (Figure 2B). The C═C (alkene) peak overlapped with the C═O peak in the acetate group at 1621 cm^−1^. The C═C (aromatic) stretching was shown at 1484 cm^−1^. The N─H and O─H stretching peaks were shifted to 3389 cm^−1^. These findings indicated that DMA formed coordination bonds on MNPs. Quantitatively, the amount of DMA coated on MNPs was 2.16 ± 0.13 mg per mg of MNPs, as measured by UV absorbance.

### 2.2 Dynamic Mechanics of COPMA‐MNP and COPMA‐MNP@DMA Hydrogels

Although the mechanical properties of light‐responsive COPMA hydrogels can be tunable by changing the COPMA concentrations under UV irradiation, other intrinsic properties (e.g., pore size) will also be simultaneously changed, which is challenging to rule out the effects on cell behaviors [32]. Strategies for improving the mechanical properties of hydrogels without significantly changing other components were developed by introducing nanoparticle crosslinkers [33, 34]. Here, we introduced carboxylic acid‐functionalized MNP as physical nanoparticle crosslinkers to COPMA hydrogels, resulting in the formation of hydrogen bonding between hydrogels and nanoparticles (Figure 1B, top). A series of 15% (w/v) COPMA hydrogels with different MNPs concentrations at 0%, 0.5%, 1% (w/v) were labeled as COPMA_15_, COPMA_15_‐MNP_0.5_, and COPMA_15_‐MNP_1_. Photocrosslinking in situ was conducted to monitor the crosslinking kinetics and evaluate the mechanical properties (Figure 2C). Before UV exposure, storage modulus (G′) was low for all polymer solutions. After UV irradiation, G′ of COPMA hydrogels increased to 331 ± 34 Pa (Figure 2C, gray symbols). Interestingly, nanocomposite hydrogels with a low MNP concentration of 0.5% (w/v) were formed with a significant increase in G′ to 1072 ± 45 Pa (Figure 2C, orange symbols), indicating MNPs acting as physical nanoparticle crosslinkers in COPMA hydrogels. When the concentration of MNP increased to 1% (w/v), G′ was 970 ± 6 Pa (Figure 2D; Figure S2A, green symbols), indicating that a higher concentration of MNP did not further contribute to the stiffness. This might be due to a trade‐off between the noncovalent bonds at the interface of COPMA and MNP and covalent bonds triggered by UV crosslinking. With increasing concentrations of MNPs, the enhanced darkness of the samples significantly restricted UV penetration, resulting in a low photocrosslinking degree in COMPA hydrogels.

To further increase the mechanical properties of the nanocomposite hydrogels, we hypothesized that chemical nanoparticle crosslinkers may contribute to hydrogel stiffness. We developed methacrylamide‐functionalized nanoparticles based on the above physical nanoparticle crosslinkers, resulting in the ability of dual‐covalent‐physical crosslinking with COPMA hydrogels under UV irradiation (Figure 1B). A series of 15% and 25% (w/v) COPMA hydrogels with different MNP@DMA concentrations at 0.5% and 1% (w/v) were labeled as COPMA_15_‐MNP@DMA_0.5_, COPMA_15_‐MNP@DMA_1_, COPMA_25_‐MNP@DMA_0.5_, and COPMA_25_‐MNP@DMA_1_, respectively. Compared to COPMA_15_‐MNP_0.5_ (1072 ± 45 Pa), the G′ of COPMA_15_‐MNP@DMA_0.5_ increased to 1130 ± 45 Pa. This slight increase may be due to the chemical nanoparticle crosslinkers (MNP@DMA) creating more physical and covalent bonds with COPMA than the physical nanoparticle crosslinkers (MNPs). Although the low concentration of nanoparticles only slightly increased the stiffness of hydrogels, the higher concentration of nanoparticles magnified this increase. Compared to COPMA_15_‐MNP_1_ (Figure 2D, green column), the G′ of COPMA_15_‐MNP@DMA_1_ significantly increased to 1328 ± 63 Pa at 1% (w/v) (Figure 2D; Figure S2A, purple symbols). This indicated that the dual covalent‐physical crosslinking between nanoparticles and COPMA compensated for the reduced UV crosslinking efficiency caused by limited UV penetration at high concentrations of black nanoparticles (Figure 1B, below). Besides, increasing the concentration of COPMA from 15% to 25% significantly improved the mechanical properties of the hydrogels up to 10‐fold of stiffness. Specifically, G′ of COPMA_25_‐MNP_0.5_ increased to 11 ± 0.047 kPa (Figure S2B,C, pink symbols) and G′ of COPMA_25_‐ MNP@DMA_0.5_ increased to 11.4 ± 0.5 kPa (Figure S2C, blue symbols). This indicated that at low concentrations of MNPs, COPMA concentrations dominate the stiffness of the nanocomposite hydrogels due to the ability to form more covalent bonds and a more condensed hydrogel network.

As chemical nanoparticle crosslinkers did not significantly contribute to the hydrogel stiffness, we further investigate the effects of crosslinking on the mechanical dynamics (e.g., stress‐relaxation) of the hydrogel network, which can significantly modulate cell behaviors [22]. Recently, hydrogels with tunable stress‐relaxation have been widely developed. However, stiffness is usually simultaneously changed, which makes it challenging to rule out the effect of stiffness from the effect of stress‐relaxation on cell behaviors. Herein, we investigated the stress‐relaxation properties of three different hydrogels, including COPMA hydrogels alone, physically crosslinked nanoparticles (MNPs) containing COPMA hydrogels, and chemically crosslinked nanoparticles (MNP@DMA) containing COPMA hydrogels. The stress relaxation tests were conducted with 20% strain (Figure 2E). Compared to the rapid stress relaxation rate of soft COPMA_15_ hydrogels (Figure 2E,F, gray symbols), the stiffer COPMA_15_‐MNP_0.5_ hydrogels showed a slower stress relaxation rate but without a significant difference (Figure 2F, orange column), probably due to the dynamic noncovalent bonds between MNP and COPMA (Figure 1B, top). Interestingly, the relaxation time of COPMA_15_‐MNP@DMA_0.5_ significantly increased (Figure 2F, blue column), indicating that the covalent photocrosslinking between MNP@DMA and COPMA reduced the dynamics of the hydrogel network (Figure 1B, below). Overall, we verified that the mechanical properties of the nanocomposite hydrogels could be tuned by simply changing the surface coatings of MNPs without significantly changing other components.

Additionally, many tissues (such as ligaments, tendons, and muscles) can mechanically adapt in a dynamic environment during daily activities [35]. To assess the adaptability of hydrogels to dynamic environments and their potential for mimicking ligaments, we tested the self‐healing properties of these hydrogels. First, we conducted a strain sweep from 0.1% to 500%. The hydrogel networks were broken down at 312% ± 34% strain (COPMA_15_), 217% ± 20% strain (COPMA_15_‐MNP_0.5_), and 177% ± 14% strain (COPMA_15_‐MNP@DMA_0.5_), respectively (Figure S2F,G). Then, cyclic strain tests at 1% and 500% strains were conducted. All hydrogel networks deformed at high strain (500%) and rapidly recovered to their initial value at low strain (1%) (Figure 2G). When a nanocomposite hydrogel was broken, two pieces of hydrogel reintegrated spontaneously, which could sustain a certain strain without separation (Figure 2H). The self‐healing process of COPMA‐MNP and COPMA‐MNP@DMA might involve disconnection and reconnection of both hydrogen bonds and coordination bonds (Figure 1B; Figure S2H). Both covalent and non‐covalent crosslinking contributed to the mechanical property of hydrogels. Many non‐covalent crosslinking sites were present in COPMA hydrogel and on the surface of the nanoparticles, forming hydrogen bonding and metal‐coordination bonding, which could significantly complement the loss of covalent bonds. The storage modulus of the nanocomposite hydrogels was approximately 3‐fold higher than that of COPMA hydrogels (Figure 2D), indicating the large contribution of non‐covalent bonds to the hydrogels. These findings were consistent with some existing self‐healable hydrogels with both covalent and non‐covalent bonds. For example, Chen et al. observed similar self‐healing behavior in the nanocomposite hydrogels after high shear in continuous step strain rheological tests [36]. Qi et al. demonstrated the self‐healing property of UV‐crosslinked phenylboronic acid modified methacrylated gelatin (GelMA‐PBA) and thiolated hyaluronic acid (HA‐SH) hybrid hydrogels [37]. Wang et al. reported the self‐healing property of UV‐crosslinked methacrylated gelatin (GelMA) and three‐arm methacrylated host‐guest supramolecule (HGSM) (HGGelMA) hybrid hydrogels containing both host‐guest interactions and covalent bonds [38].

### 2.3 Adaptable and Stable Magnetic‐Driven Alignment

Considering the inherent anisotropic structures of ligaments, matrix with anisotropic topography plays an important role in guiding cell growth, ECM production, and tissue regeneration [39]. To develop anisotropic hydrogels, we applied a straightforward method by parallelly applying two commercial magnets among two sides of the hydrogel precursors under UV irradiation (Figure 2I). After applying a magnetic field (35 ± 2 mT) for 1–5 s, magnetic nanoparticles started to assemble oriented, and this organized topography was more obvious after 10 s of magnetic field application time, indicating its rapid magnetic response (Figure 2J). Besides, these nanocomposite hydrogel precursors can be injected into irregular sites, then rapidly form hydrogels with anisotropic morphology that could adapt to irregular shapes with the combination of an external magnetic field and UV irradiation (Figure 2I; Figure S3). These properties may enable the injection of the hydrogel precursors with numerous cells and biophysical anisotropic cues to the irregular wound with rapid magnetic and UV stimulation, which may facilitate rapid cell recruitment, alignment, and differentiation.

After the fabrication of anisotropic hydrogels, they were soaked in the cell culture medium for 28 days. Optical microscopy images showed that the chemical/physical nanoparticle crosslinkers at a concentration of 0.5% (w/v) were homogeneously aligned parallel to the magnetic field direction (Figure 2K). In the absence of the magnetic field, the nanoparticles were randomly dispersed in the hydrogel network (Figure S4). Besides, COPMA_25_‐MNP_0.5_ hydrogels with a dense network showed a homogenous aligned structure as COPMA_15_‐MNP_0.5_ hydrogels (Figure S5). However, when the concentration of the nanoparticle crosslinkers increased to 1% (w/v), the nanoparticles aggregated in the center of hydrogels (Figure S5). These findings demonstrated the homogenous anisotropic hydrogels depend on the concentration of magnetic nanoparticles instead of polymer concentration, indicating that this rapid magnetic‐light stimulation method could be an effective and universal method to produce various aligned hydrogels. Besides, anisotropic magnetic nanocomposite hydrogels could be scaled up from droplet (6.8 mm*4 mm*2 mm, length*width*height) to large hydrogels (e.g., 10.4 mm*2 mm, diameter*height and 39 mm*7.5 mm*2 mm, length*width*height, Figure S6). These results indicated that magnetic nanocomposite hydrogels could be easily scaled up, enabling the customization of hydrogel dimensions to meet requirements of clinically relevant tissue engineering applications.

Next, we tested the anisotropic stability of hydrogels, which is essential to tissue regeneration, especially for tissues that require weeks to months for reconstruction (e.g., ligaments, tendons, and muscles) [40]. First, we obtained a brief understanding of hydrogels’ stability by imaging their morphologies in medium for 28 days. The COPMA_15_‐MNP_0.5_ and COPMA_15_‐MNP@DMA_0.5_ hydrogels kept anisotropic structures for 28 days (Figure 2K). Besides, these nanoparticle chains remained oriented after 28 days (Figure S7A). These findings demonstrated both chemical/physical nanoparticle crosslinkers could produce anisotropic nanocomposite hydrogels in a simple step with high efficiency (only exposed to the magnetic field during gelation for 3 min) and good stability. The good anisotropic stability of nanocomposite hydrogels may provide the foundation of anisotropic cell networks and ECM.

To statically study the stability of hydrogels, gel fractions and swelling ratios of hydrogels were quantified (Figure S7B–D). The gel fraction of nanocomposite hydrogels remained at a higher level (∼80%) than that of COPMA_15_ hydrogels after 1 and 28 days (Figure S7B). Swelling ratios of nanocomposite hydrogels were significantly lower than that of COPMA_15_ hydrogels on day 1 and significantly increased on day 28 (Figure S7D). Overall, introducing low concentration of magnetic nanoparticles (0.5% w/v) could efficiently endow COPMA‐based hydrogels with magnetic‐light‐response, anisotropic morphology, and good stability.

### 2.4 Biocompatibility of hMSCs Laden Hydrogels

**FIGURE 3:**
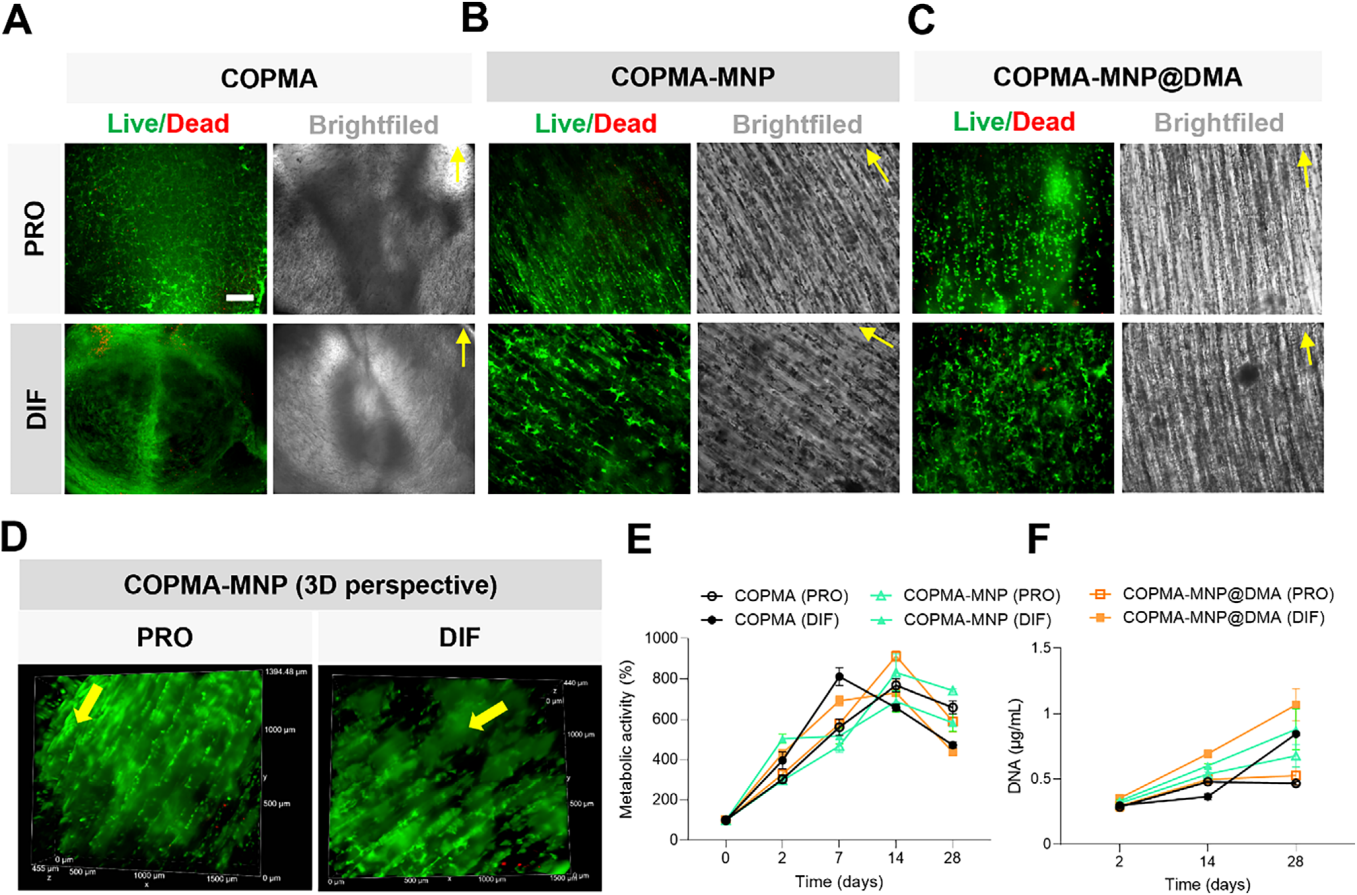
Biocompatibility of hMSCs laden COPMA, COPMA‐MNP, and COPMA‐MNP@DMA hydrogels up to 28 days. (A–C) Live/dead staining of hMSCs laden hydrogels in proliferation and differentiation media on day 28, scale bar: 200 µm. (D) Live/dead staining images of COPMA‐MNP constructs in 3D perspective. Yellow arrows indicate the magnetic field direction. (E) Metabolic activity. (F) DNA content in constructs (*n* = 3–4).

The COPMA_15_ hydrogels with a low concentration of nanoparticle crosslinkers (0.5% w/v) were chosen to further investigate the biocompatibility and cell‐hydrogel interaction, due to their stable homogenous anisotropic morphology, suitable stiffness, and swelling ratio (Figure 2). The biocompatibility of hMSCs laden COPMA_15_ (COPMA), COPMA_15_‐MNP_0.5_ (COPMA‐MNP), and COPMA_15_‐MNP@DMA_0.5_ (COPMA‐MNP@DMA) hydrogels was investigated by live/dead staining, metabolic activity, and DNA quantification. To assess the biocompatibility of the constructs under both proliferative and differentiative conditions, the same constructs were cultured in two types of culture media: proliferation medium (without growth factors) and differentiation medium (supplemented with transforming growth factor beta‐3). From live/dead staining images, hMSCs showed high viability in all constructs with magnetic guidance, indicating the hydrogel components did not affect cell viability (Figure 3A–D; Figure S8). Interestingly, hMSCs parallelly spread along the anisotropic guidance of both COPMA‐MNP and COPMA‐MNP@DMA hydrogels, but randomly spread in the COPMA hydrogels, although under the same magnetic field. The metabolic activity of encapsulated hMSCs in 3D constructs showed an increased trend under both proliferation and differentiation conditions, with a 4‐ to 7‐fold increase in all constructs when comparing day 0 to day 28 (Figure 3E). Besides, the DNA content in the cell‐laden hydrogels increased over time (Figure 3F). These findings indicated good biocompatibility of hydrogels with or without magnetic nanoparticles over 28 days. Overall, these nanocomposite hydrogels could induce cell alignment with good biocompatibility and stability for 28 days, regardless of hydrogel mechanics and growth factors in the media.

### 2.5 Biophysical Topography Regulates 3D Cell Alignment

**FIGURE 4:**
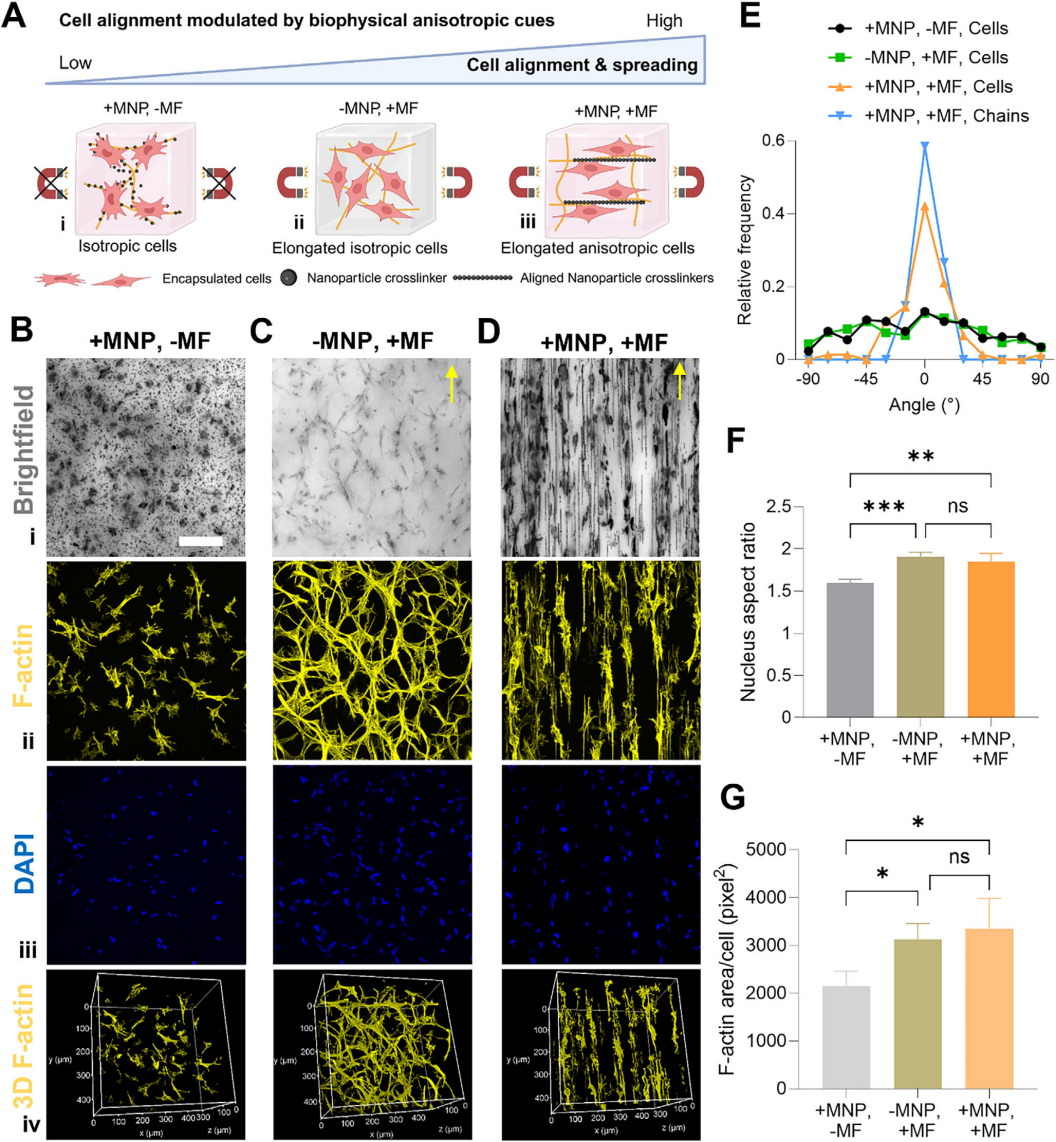
Positive effect of biophysical anisotropic cues on cell alignment. (A) Illustration of constructs with or without anisotropic cues on day 14. (B–D) Confocal images of constructs, scale bar: 100 µm. The yellow arrows indicate the direction of the magnetic field. (B) Constructs with MNPs but no MF. (C) Constructs without MNPs but with MF. (D) Constructs with both MNPs and MF. (E) Angle distribution of cells and MNP chains in constructs. (F) Nucleus aspect ratio. (G) Normalized cell spreading area (*n* ≥ 3, ns indicates no significant difference, ^*^ *p* < 0.05, ^**^ *p* < 0.01, ^***^ *p* < 0.001).

As the magnetic remote‐control strategy demonstrated its significant contribution to the formation of aligned nanoparticle chains in the above sections, we further quantitatively investigated cell spreading and alignment in the presence of biophysical anisotropic cues. Herein, we encapsulated hMSCs in the COPMA‐based hydrogels with different conditions: (i) with MNPs but without a magnetic field (Figure 4Ai), (ii) without MNPs but with/without a magnetic field (Figure 4Aii; Figure S9), and (iii) with both MNPs and magnetic field (Figure 4Aiii) to study the effect of remote‐control magnetic strategy on 3D cell alignment. In the absence of anisotropic cues, hMSCs randomly spread in nanocomposite hydrogels lacking magnetic field exposure (Figure 4Ai,B). Similarly, hMSCs exhibited random spreading within COPMA hydrogels without MNPs regardless of the magnetic field application (Figure 4Aii,C; and Figure S9). In contrast, the 3D aligned hMSCs networks were observed in the nanocomposite hydrogels under a magnetic field (Figure 4Aiii,D). The quantification results of angular distributions showed that cells were aligned (Figure 4E, orange symbol) along the anisotropic MNP chains (Figure 4E, blue symbol), but disorganized in the isotropic constructs (Figure 4E, black and green symbols). These findings indicated that the anisotropic MNP chains were critical to the formation of 3D aligned cell networks.

Interestingly, the quantification of nucleus aspect ratio and cell spreading area in the above hydrogels showed differences as well (Figure 4F,G). Specifically, the nucleus aspect ratio and cell spreading area in COPMA hydrogels alone (Figure 4F,G, dark yellow columns) were significantly larger than in COPMA‐MNP hydrogels without anisotropic cues (Figure 4F,G, gray columns), suggesting that the soft COPMA matrix facilitated cell spreading. Besides, nucleus aspect ratio and hMSCs spread area in the nanocomposite hydrogels with anisotropic cues (Figure 4F,G, orange columns) were significantly higher than that without anisotropic topology (Figure 4F,G, gray columns), indicating oriented topography facilitated cell spreading. Moreover, the nucleus aspect ratio and normalized F‐actin spreading area in soft COPMA hydrogels were similar as that in anisotropic stiff COPMA‐MNP hydrogels (Figure 4F,G), demonstrating that anisotropic cues could compensate for the negative effect of high stiffness on cell spreading. Overall, 3D aligned cell networks benefited from anisotropic biophysical cues created by a magnetic remote‐control strategy.

### 2.6 Biomechanical Cues Modulate 3D Cell Alignment

**FIGURE 5:**
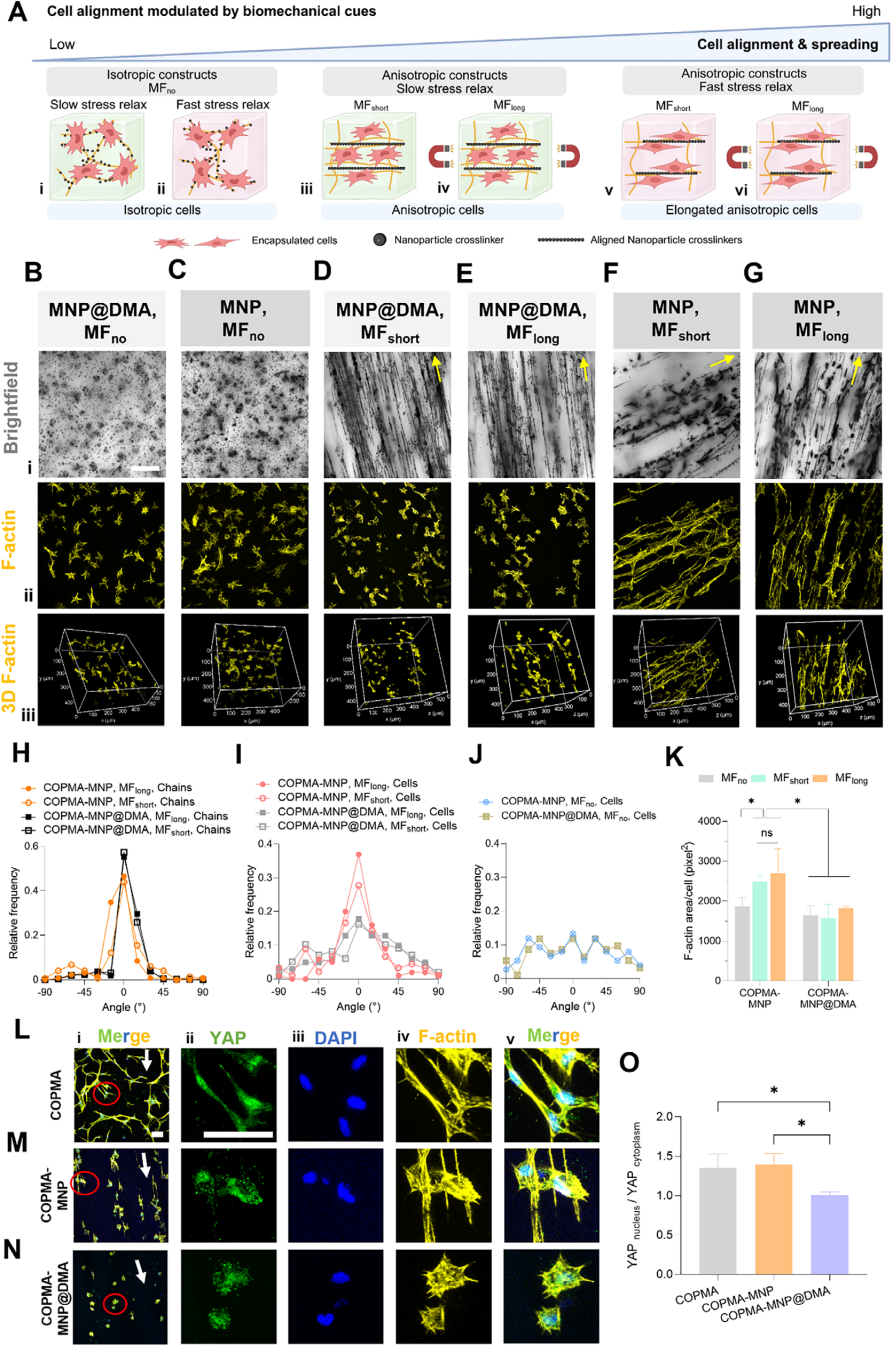
Effect of hydrogel mechanics on cell alignment. (A) Scheme of single and combined effects of biomechanical and biophysical anisotropic cues on cell alignment on day 28. (B–G) Confocal images of constructs, scale bar: 100 µm. Yellow arrows indicate the direction of the magnetic field. (H) Angle distribution of MNP and MNP@DMA chains under the magnetic field. (I) Angle distribution of encapsulated cells in COPMA‐MNP and COPMA‐MNP@DMA constructs under the magnetic field. (J) Angle distribution of encapsulated cells in constructs without the magnetic field. (K) Normalized F‐actin spreading area. (L–N) YAP staining images in COPMA, COPMA‐MNP, COPMA‐MNP@DMA cell‐laden hydrogels with MF_short_, scale bar: 50 µm. (i) Merged overview images. (ii–v) magnified regions corresponding to the red circles in (i) images. White arrows indicate the direction of the magnetic field. (O) Comparison of YAP nuclear location (*n* ≥ 3, ns indicates no significant difference, ^*^ *p* < 0.05).

After showing that the stiffness of the developed hydrogels affects cell spreading, we further modulate cell spreading and alignment by stress relaxation rates. Herein, hMSCs were encapsulated in the physically crosslinked hydrogels with fast stress‐relaxation (COPMA‐MNP hydrogels) or chemically crosslinked hydrogels with slow stress‐relaxation (COPMA‐MNP@DMA hydrogels) for 28 days (Figure 5A). In addition, to assess the efficiency of this magnetic strategy, constructs were exposed to different magnetic field application times: 0 min, 3 min during gelation, or continuously for 24 h × 28 days during cell culture, referred to as MF_no_, MF_short_, and MF_long_, respectively. Nanoparticles randomly distributed in isotropic constructs without MF regardless of hydrogel mechanics (Figure 5B,Ci). However, both MNP@DMA (Figure 5Di,Ei) and MNP chains (Figure 5Fi,Gi) kept oriented in the constructs with MF (Figure 5H), indicating hydrogel mechanics and cell encapsulation did not interrupt the anisotropic structures. Among these anisotropic constructs, the duration of the magnetic field (MF_short_ and MF_long_) showed a similar effect on the alignment of the nanoparticle chains (Figure 5H). Interestingly, as we expected, nanoparticle chains were more homogeneously formed in chemically crosslinked hydrogels (Figure 5H, black symbols) under the external magnetic field compared with physically crosslinked hydrogels (Figure 5H, orange symbols). This may be due to the stronger interactions between chemically crosslinked nanoparticles and hydrogels (Figure 1B), resulting in nanoparticles being frozen in the hydrogel network even during cell culture for 28 days.

Moreover, cells in chemically crosslinked hydrogels under anisotropic signals (Figure 5I, gray symbols) were slightly more aligned than cells in chemically/physically crosslinked hydrogels without alignment (Figure 5J), but less aligned compared to cells in physically crosslinked hydrogels under anisotropic cues (Figure 5I, pink symbols). Furthermore, (3D) F‐actin staining results showed that cells less spread in the constructs with chemically crosslinked nanoparticles (Figure 5D,Eii,iii) than physical crosslinked nanoparticles (Figure 5F,Gii,iii) even under biophysical anisotropic guidance. Among the isotropic constructs, cell distribution (Figure 5J) and spreading area (Figure 5K, gray columns) did not significantly improve in the constructs with faster stress relaxation, indicating biomechanical cues itself did not influence cell alignment and spreading. However, cells spread more in physically crosslinked anisotropic hydrogels with fast stress‐relaxation (Figure 5K, left) than in chemically crosslinked anisotropic hydrogels with slow stress relaxation (Figure 5K, right). Additionally, compared to anisotropic constructs with MF_short_, the longer duration of the magnetic field (MF_long_) did not result in a significant improvement in alignment of cell network (Figure 5I) and cell spreading (Figure 5K, light blue and orange symbols). This indicated that the magnetic‐remote control strategy was efficient for fabricating anisotropic cell networks even without external continuous magnetic guidance during cell culture. Overall, these results indicated matrix mechanical dynamics at similar stiffness levels did not significantly influence cell spreading and alignment. However, constructs with fast stress relaxation with the assistance of biophysical anisotropic cues facilitated cell organization and spreading.

To reveal the mechanism, we compared the nuclear localization of Yes‐associated protein (YAP, a key regulator of cells in responding to biomechanical cues) in hydrogels with different stress relaxation rates [41]. In the fast stress relaxed hydrogels (COPMA and COPMA‐MNP), encapsulated cells spread well (Figure 5L,M), and YAP was localized more in the nucleus than in the cytoplasm (Figure 5O, gray and orange columns). However, in the slow stress relaxed hydrogel (COPMA‐MNP@DMA), encapsulated hMSCs remained rounded (Figure 5N), and YAP expression was at a similar level in the nucleus and the cytoplasm (Figure 5O, purple column). Encapsulated cells were aligned in the COPMA‐MNP and COPMA‐MNP@DMA hydrogels but not in COPMA hydrogels, indicating stress relaxation itself did not influence cell orientation. We hypothesized that these differences are likely because cells generated contractile forced during culture, which could be dissipated faster in hydrogels with fast stress relaxation rate than those with a slower stress relaxation rate. These forces transduced in the fast stress relaxed hydrogel increase cell‐matrix interactions and ligand clusters, facilitating cell spreading and YAP nuclear localization [22]. The enhanced cell‐matrix interaction and cell spreading may further exert positive effects with biophysical anisotropic and biochemical cues to facilitate the deposition of bioactive proteins and cell differentiation.

### 2.7 Biochemical or/and Biophysical Cues Modulate Cell Alignment and Differentiation

Next, we assessed the solo and combined effects of biochemical cues (e.g., growth factors), biophysical anisotropic cues (hydrogel topography), and biomechanical cues (hydrogel dynamics) on cell differentiation and alignment. We evaluated cell differentiation by the expression of collagen type I (COL‐I) and scleraxis (SCX), two representative markers for ligamentogenic/tenogenic differentiation [42]. To assess the effect of biochemical cues on cell differentiation, we cultured the same constructs in two culture conditions: differentiation condition (with TGF‐β3, a ligament/tendon inducing growth factor) [43], and proliferation condition (without TGF‐β3) for 28 days.

**FIGURE 6:**
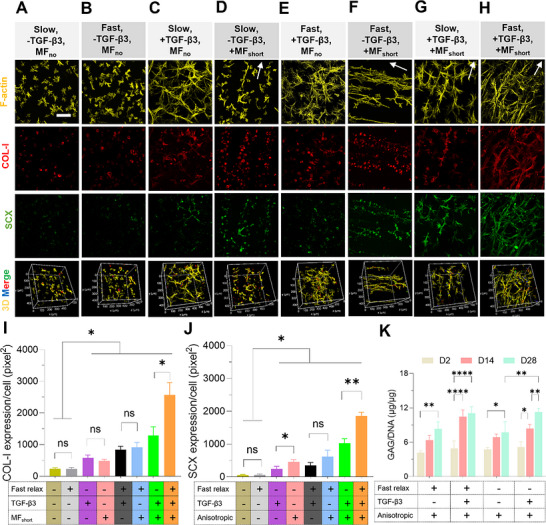
The solo and combined effects of biomechanical, biochemical, and biophysical anisotropic cues on cell alignment and ligamentogenic/tenogenetic differentiation. (A–H) Immunostaining images of constructs on day 28. Constructs were cultured without a magnetic field (MF_no_) or with a magnetic field once for 3 min during gelation (MF_short_) with or without TGF‐β3 (±TGF‐β3). Scale bar: 100 µm. White arrows indicate the direction of the magnetic field. (I,J) Comparison of COL‐I and SCX expressions in constructs (*n* ≥ 3). (K) Normalized GAG/DNA production in anisotropic constructs (*n* ≥ 3, ns indicates no significant difference, ^*^ *p* < 0.05, ^**^ *p* < 0.01, ^****^ *p* < 0.0001).

First, we assessed the solo effect of biomechanics cues on cell differentiation. Comparable levels of COL‐I and SCX expression were observed in isotropic nanocomposite constructs with either slow (Figure 6A,I,J, dark yellow columns) or fast (Figure 6B,I,J, gray columns) stress relaxation rates in the absence of growth factors. This suggested that biomechanical cues alone did not significantly influence cell differentiation.

Second, the solo effect of biochemical cues on cell differentiation and alignment was evaluated. More COL‐I and SCX were expressed under differentiation conditions (Figure 6C,I,J, purple columns) than proliferation conditions (Figure 6A,I,J, dark yellow columns) in the isotropic nanocomposite constructs, indicating that TGF‐β3 alone promoted cell differentiation toward ligamentocytes/tenocytes. Cells’ spreading area was significantly higher in the isotropic nanocomposite constructs cultured with TGF‐β3 than those without TGF‐β3 (Figure S10), indicating the positive effect of biochemical cues on cell spreading. In the presence of TGF‐β3, cell networks were aligned in the anisotropic constructs (Figure S11, green and orange symbols), but disorganized in the isotropic constructs (Figure S11, purple and gray symbols), in line with the alignment trend in constructs without growth factors (Figure 5I,J). This indicated that biochemical cues alone did not significantly affect cell alignment.

Third, we assessed the solo effect of biophysical anisotropic cues on cell differentiation and alignment. COL‐I and SCX expressions were significantly higher in anisotropic nanocomposite constructs (Figure 6D,I,J, pink columns) than in isotropic nanocomposite constructs (Figure 6A,I,J, yellow columns) in the absence of growth factors. Besides, cells spread and aligned in the anisotropic constructs instead of in the isotropic constructs (Figure S11). These findings demonstrated that aligned constructs could promote hMSCs differentiation and orientation. Interestingly, the isotropic constructs with TGF‐β3 (Figure 6I,J, purple and black columns) exhibited similar COL‐I and SCX expression levels to the anisotropic constructs in the absence of TGF‐β3 (Figure 6I,J, pink and blue columns). This suggested that anisotropic cues alone exhibited a comparable cell differentiation‐inducing effect to the biochemical cues in non‐aligned constructs.

Next, we hypothesized that the combined effects of the biochemical and biophysical cues implemented in our hydrogels might synergistically influence cell alignment and differentiation. First, we assessed the combined effect of biochemical and biomechanical cues on cell behavior. COL‐I and SCX production were significantly higher in the isotropic COPMA‐MNP constructs with TGF‐β3 (Figure 6E,I,J, black columns) than isotropic COPMA‐MNP@DMA with/without TGF‐β3 (Figure 6A,C,I,J, yellow and purple columns), indicating that biochemical and biomechanical cues complementarily promoted cell differentiation.

Second, we assessed the combined effects of biophysical anisotropic and biomechanical cues on cell behavior. Higher amounts of COL‐I and SCX were observed in the anisotropic constructs with a fast stress relaxation rate (Figure 6F,I,J, blue columns) than isotropic (Figure 6A,I,J, yellow columns) or anisotropic constructs (Figure 6D,I,J, pink columns) with slow stress relaxation rates in absence of growth factors. This indicated that aligned constructs with faster stress relaxation rate could amplify the positive effect of cell alignment on cell differentiation.

Third, we assessed the combined effect of biochemical cues and biophysical anisotropic cues on cell behavior. COL‐I and SCX production were significantly higher in the anisotropic COPMA‐MNP@DMA constructs with TGF‐β3 (Figure 6G,I,J, green columns) than isotropic COPMA‐MNP@DMA constructs without TGF‐β3 (Figure 6A,I,J, yellow columns), indicating that biochemical and biophysical anisotropic cues complementarily promoted cell differentiation.

Finally, the combined effects of biochemical and biophysical cues on cell behavior were determined. Significantly higher amounts of COL‐I (2‐fold) and SCX (1.8‐fold) were observed in the anisotropic constructs with fast stress relaxation rate (Figure 6H,I,J, orange columns) than that with slow stress relaxation rates (Figure 6G,I,J, green columns) with the assistance of growth factors. These 3 stimulation cues showed higher cell alignment and differentiation effects than the above single cues or combinations of cues, indicating the synergistic effect of fast stress relaxation, growth factors, and anisotropic topography further facilitated cell alignment and differentiation. Interestingly, we found that short‐term magnetic actuation (only 3 min during gelation, MF_short_) yielded comparable cell orientation and differentiation effects as long‐term magnetic actuation (28 days during cell culture, MF_long_, Figure S12). This indicated that achieving and regulating cell alignment and fate within our MNP‐laden hydrogel models were both highly efficient and effective.

As one of the most abundant ECM components in connective tissues, glycosaminoglycans (GAGs) were quantified to represent ECM production in constructs. The normalized GAG amount rose in all anisotropic (Figure 6K) and isotropic constructs (Figure S13) over 28 days. Specifically, a significant increase GAG/DNA from day 2 to day 14 was shown in anisotropic COPMA‐MNP and COPMA‐MNP@DMA constructs in differentiation medium (Figure 6K), but not in isotropic constructs (Figure S13A,B), indicating that the anisotropic construct facilitated GAG production. In COPMA‐MNP@DMA constructs, significantly higher GAG/DNA was observed in differentiation conditions than in proliferation conditions, regardless of constructs morphology (Figure 6K, right, and Figure S13B), indicating the positive effect of biochemical cues on GAG production. However, in anisotropic COPMA‐MNP constructs, no significantly higher GAG production was detected under differentiation conditions compared to proliferation conditions, indicating the positive complementary effect of biophysical anisotropic cues and fast stress relaxation on GAG production even without stimulation of biochemical cues (Figure 6K, left). Overall, constructs with anisotropic guidance, fast stress relaxation, and biochemical cues facilitated oriented cellular networks, differentiation, and ECM production.

## 3 Discussion

Hydrogels are widely developed as artificial ECMs and have the potential to be used in in vitro 3D models to investigate cell‐ECM interactions [44]. Hydrogels not only provide a 3D biophysical network but also offer a dynamic environment capable of transporting biochemical cues to modulate the behavior of encapsulated cells [45]. However, anisotropic ECM‐mimicking natural hydrogels remain less developed, because bulk hydrogels typically lack oriented structures. Besides, cell behaviors in cell‐laden hydrogels are highly affected by biophysical cues (e.g., topography), biophysical mechanics (e.g., hydrogel stiffness and viscoelasticity), and biochemical cues (e.g., growth factors) [45]. Yet, a systematic modulation of the above cues to direct 3D cell alignment and cell fate in cell‐laden hydrogels is still limited. Herein, we aim to develop a 3D in vitro model by employing novel anisotropic nanocomposite hydrogels with tunable mechanical stiffness and dynamics to modulate encapsulated cell alignment and differentiation by the combination of biophysical and biochemical triggers.

In this study, a dual magnetic‐light‐responsive nanocomposite hydrogel was fabricated with an oriented structure, self‐healing properties, tunable storage modulus, and stress relaxation rates. COPMA hydrogels have been developed with rapid UV crosslinking properties but lack uniaxial topography [32]. MNPs could endow hydrogels with an oriented structure and increase their mechanical properties by acting as nanoparticle fillers or physical crosslinkers to increase crosslink density [46]. However, MNPs typically form noncovalent bonds with hydrogels instead of covalent bonds, resulting in less control over the nanocomposite hydrogels [47]. MNPs could be coated with various functional groups to obtain versatile properties, such as amine, carboxylate, and catechol groups [31, 48]. Herein, a dual magnetic‐light‐responsive nanoparticle crosslinker was developed by functionalizing MNPs with DMA to enhance both physical and chemical properties of COPMA hydrogels (Figure 1A,B). Compared to COPMA hydrogels, nanocomposite hydrogels showed improved stiffness even with a low concentration of nanoparticles (0.5% w/v). Importantly, we prepared COPMA‐based hydrogels with different mechanical dynamics by simply modifying the MNP coating without changing other components. The stress relaxation rate of COPMA‐MNP@DMA hydrogel was significantly lower than COPMA and COPMA‐MNP hydrogels, due to the formation of both covalent and noncovalent networks between chemical nanoparticle crosslinkers and COPMA. These COPMA‐based hydrogels with different stiffness and stress relaxation rates may serve as a promising platform for modulating cell behaviors in situ.

Magnetic‐responsive cell‐laden hydrogels, such as magnetic cells, magnetic fibers, and MNPs laden hydrogels are attracting more attention in modulating cell alignment or/and differentiation [49, 50, 51, 52]. However, when we aim to fabricate 3D aligned magnetic‐responsive cell‐laden hydrogels, magnetic cell/fiber‐laden hydrogels typically require complicated procedures, such as endocytosis of MNPs into cells or electrospinning and cryosection of magnetic fibers in advance. Thus, directly mixing MNPs, cells, and hydrogel precursors is a more straightforward approach to develop anisotropic cell‐laden matrices in situ [53]. However, different from magnetic cell/fiber‐laden constructs, it is more challenging to induce long‐term 3D cell alignment by directly aligned MNP chains in hydrogels, probably due to the limited interaction between MNPs and cells. Many studies have successfully generated aligned MNP chains within cell‐laden hydrogels, yet failed to induce corresponding 3D cell alignment, highlighting the gap between constructing aligned MNP chains and aligned 3D cell networks [54]. Furthermore, while some reports have demonstrated short‐term cell alignment in MNP‐laden hydrogels (typically 2–7 days), the persistence of cell alignment and bioactivity over longer periods is poor [55, 56]. Collectively, despite existing extensive efforts, no previously reported studies achieved and modulated 3D cell alignment and differentiation in long period (i.e., for at least 28 days) by directly encapsulating MNPs into cell‐laden hydrogels. In this study, we developed a simple approach to fabricate stable 3D aligned cell networks in hydrogels with MNPs in the presence of a mild magnetic field (35 ± 2 mT) and light irradiation (365 nm, 20 mW·cm^−2^). Our nanocomposite hydrogels showed rapid magnetic response in 20–60 s, and rapid UV‐response in 1–3 min. Interestingly, the anisotropic nanoparticle chains could be adaptable to the irregular‐shaped container. This simple magnetic‐remote control strategy may hold great potential in minimally invasive surgery by injecting a hydrogel precursor with cell suspensions, followed by application of an external magnetic field and rapid photocuring. We verified that both MNP chains and 3D cell networks remained aligned and stable for 28 days in oriented nanocomposite constructs, even without a magnetic field during cell culture. Besides, we demonstrated that short‐term magnetic actuation (only 3 min during gelation at once) yielded comparable cell orientation and differentiation effects as long‐term magnetic actuation (28 days during cell culture). This indicates that achieving and regulating cell alignment and fate within our MNP‐laden hydrogel models are both highly efficient and effective.

UV irradiation, magneto‐mechanical effect, and MNP cell uptake have been revealed with possible cytotoxicity [57, 58, 59, 60, 61]. Cytotoxicity of UV irradiation was time‐dependent and dose‐dependent [57, 58, 62, 63, 64]. For example, Nguyen et al. and Bennet et al. revealed that longer UV exposure induced increasing cell damage at the same UV intensity [57, 58]. Shorrocks et al., Godar et al. and McMillan et al. demonstrated that higher UV irradiation dose over the same duration caused lower cell viability [62, 63, 64]. In this study, cell‐laden hydrogels were gelated under mild UV light (365 nm, 20 mW·cm^−2^) for 3 min, since it has shown adequate hMSC cytocompatibility in our previous study, indicating the negligible cytotoxicity from mild UV irradiation in our 3D hydrogel system [32]. Magneto‐mechanical effect can also induce cell death by physically disturbing cellular structures under a dynamic magnetic field (oscillating, rotating, or gradient), inducing MNPs movement (vibration, torque, or translation) [59, 60]. Thebault et al. demonstrated that the longer exposure time (1–60 min) under 20 Hz rotating magnetic field or higher frequency of rotating magnetic field (2–20 Hz) for 40 min led to lower cell viability, but no significant cytotoxicity was observed under the same strength of static magnetic field (600 mT) for the same magnetic exposure time (1–60 min) [65]. This demonstrated that both dynamic magnetic field and MNPs movement are essential to induce magneto‐mechanical effect. In our study, MNPs were rapidly assembled into aligned MNP chains in 10 s under a mild magnetic field (35 ± 2 mT) (Figure 2J), which is 12 times lower than the magnetic field of 410 mT in Chio et al. study and 3 times lower than magnetic field of 100 mT in Rose et al. study [66, 67]. These aligned MNP chains were fixed in the hydrogel network under a 3 min simultaneous exposure of UV and static magnetic field. After 3 min gelation, UV and static magnetic field were removed, and no significant movement of MNP chains was observed (Figure 2K). This means that the necessary conditions for generating the magnetic field effect (dynamic magnetic field and MNPs movement) were lacking during the cell culture period. Besides, the phagocytosis of MNPs by hMSCs could induce dose‐dependent cytotoxicity in 2D environment [61]. Our group previously revealed that 2D cultured hMSCs could phagocytose MNP in 24 h to induce 16%–24% lactate dehydrogenase (LDH) released, indicating low cytotoxicity of MNPs (≥ 75% cell viability) at different MNP concentrations (0.005–0.5% w/v) [61]. However, different from 2D cell culture system, interaction between cells and MNPs was much lower in 3D nanocomposite constructs due to the interaction between the hydrogel and MNPs, leading to more than 95% viability when MNP concentration was 0.5% w/v [61]. This indicated the low possibility of encapsulated hMSCs endocytosis MNPs in the 3D hydrogel system. Importantly, cytotoxicity related to cellular oxidation stress and inflammation is typically triggered only when endocytosed MNPs reach sufficiently high intracellular levels [68]. However, in the low cell‐MNP interaction system (e.g., MNP‐laden hydrogels), cytotoxicity of MNPs are typically lower likely due to the limited endocytosed MNPs [69, 70, 71]. Similarly, in our 3D hydrogel system, MNPs were integrated within hydrogels by non‐covalent and covalent bonds and aligned into micron‐sized MNP long chains, leading higher difficulty for hMSCs phagocytose MNP chains and low potential cytotoxicity. Overall, we observed high 3D cell viability, increasing metabolic activity of hMSCs, and increasing DNA content in 3D aligned nanocomposite constructs for 28 days (Figure 3). These findings indicated no significant negative effect from UV, magneto‐mechanical effect, or MNPs internalization under our experimental conditions (mild UV exposure: 365 nm, 20 mW·cm^−^^2^, 3 min; static magnetic field: 35 ± 2 mT; low MNP concentration: 0.5% w/v). Besides, superior to the 2D single layer cell culture, our 3D aligned nanocomposite cell‐laden hydrogels provided a biocompatible and protective microenvironment that supported cell proliferation while buffering external harm and stabilizing the aligned MNP chains.

The effect of hydrogel stress relaxation on cell spreading and differentiation has been revealed in many studies [21, 22, 72, 73, 74]. However, the interactions between hydrogel stress relaxation, encapsulated stem cell alignment, and fate remain poorly understood. No report showed the modulation of aligned cellular behavior and fate by systematically adjusting the viscoelasticity of hydrogels, alignment of MNP chains, and biochemical signal in MNP‐laden hydrogels. Although Babu et al. tried to modulate cell alignment by adjusting the stiffness of microgels rather than the stress relaxation of an entire hydrogel, they observed no significant effect on cell alignment or elongation [75]. This is probably because different stiffness of microgels did not significantly influence the overall mechanical properties of the bulk hydrogel, which the encapsulated cells primarily respond to [75]. Besides, the combination of biophysical and biochemical cues on cell behavior and fate is unknown or hard to predict based on the existing reports about MNP constructs, due to the challenge to maintain 3D cell alignment for a long period of culture (e.g., at least 28 days) [55, 56]. Our study first systematically modulated these biophysical and biochemical cues to modulate the encapsulated cell alignment, spreading, and differentiation in the MNP‐laden hydrogels. We found that the encapsulated hMSCs showed similar random spreading and low differentiation capability in isotropic COPMA‐MNP and COPMA‐MNP@DMA constructs, which showed comparable stiffness but significantly different mechanical dynamics, indicating that the biomechanical cues did not contribute themselves to cell alignment and differentiation. More cell alignment and ligamentocytes/tenocytes were observed in anisotropic COPMA‐MNP hydrogels with fast stress relaxation than in anisotropic COPMA‐MNP@DMA hydrogels with slow stress relaxation. This indicated that the anisotropic constructs with fast stress relaxation amplified the positive effect of cell alignment on cell differentiation toward ligamentocytes/tenocytes.

Moreover, we modulated the orientation and differentiation of encapsulated hMSCs in hydrogels by anisotropic biophysical and biochemical cues. Compared to constructs without TGF‐β3, higher COL‐I and SCX expressions were observed in constructs with TGF‐β3, but cell alignment was similar in the same type of constructs with/without TGF‐β3. This indicated that biochemical cues alone facilitated cell differentiation regardless of construct morphology and mechanics. By using our simplified anisotropic technique, a more oriented 3D cell network and more ligamentocytes/tenocytes were observed in our 3D anisotropic models compared to isotropic constructs without growth factors, indicating the positive effect of biophysical anisotropic cues on cell alignment and differentiation. Compared to isotropic constructs with TGF‐β3 stimulation, the anisotropic constructs without TGF‐β3 showed similar ability to promote cell differentiation, indicating a similar positive effect of biophysical anisotropic cues and biochemical cues on cell differentiation. When the biophysical anisotropic, biomechanical, and biochemical cues were combined, we found their synergistic effect on hMSCs alignment and differentiation toward ligamentocytes/tenocytes.

## 4 Conclusion

This study provided a straightforward approach to fabricate nanocomposite hydrogels with 3D aligned cell networks by rapidly employing dual magnetic‐light stimuli to systematically modulate stem cell alignment and fate in situ. We developed two types of nanoparticles crosslinkers: one based on physical crosslinkers with only magnetic‐responsiveness, the other one based on chemical crosslinkers with magnetic‐ and light‐responsive properties. The COPMA‐MNP@DMA hydrogels with chemical nanoparticle crosslinkers showed a slower stress relaxation rate due to the formation of both covalent and non‐covalent bonds within the hydrogels, compared to COPMA‐MNP hydrogels with physical nanoparticle crosslinkers. Moreover, all these 3D aligned hydrogels showed rapid UV curing and self‐healing properties, good stability, and biocompatibility for 28 days. The anisotropic constructs promoted hMSCs differentiation to ligamentocytes/tenocytes and secreted more GAG, COL‐I, and SCX when compared to isotropic constructs. Furthermore, we demonstrated that anisotropic hydrogels with fast stress‐relaxation can facilitate cell alignment and differentiation by combining biophysical anisotropic cues with biomechanical dynamics. Additionally, biochemical cues such as growth factors synergize with anisotropic cues, promoting cell differentiation and helping counteract the inhibition of cell organization in matrices with slow stress relaxation. Overall, we enabled systematic modulation of cell behaviors (e.g., cell alignment and differentiation) by employing an anisotropic 3D in vitro model in combination with biophysical topography, biomechanical, and biochemical cues. This attractive biofabrication and modulation strategy may pave the way to fabricate 3D organized constructs for various anisotropic tissue models (e.g., ligament, tendon, muscle, and nerve) with the assistance of biophysical and biochemical signals.

## 5 Experimental Section

### 5.1 Reagents

All reagents were purchased from Sigma–Aldrich and used as received, unless specifically stated below.

### 5.2 Synthesis of MNP

MNPs were synthesized according to the protocol reported by Chen et al. [33]. Briefly, ferric chloride hexahydrate (1.8 g) and sodium acetate (2.04 g) were dissolved in ethylene glycol (70 mL) under vigorous magnetic stirring for 30 min. This mixture solution was further hydrothermally reacted at 200°C for 12 h. After cooling down to room temperature, MNPs were collected by a magnet and then washed 3 times with water and ethanol, respectively. The purified MNPs were stored in ethanol at 4°C until use.

### 5.3 Synthesis of MNP@DMA

MNPs (10 mg) were suspended in tris(hydroxymethyl)aminomethane buffer (Tris buffer, pH 8.5, 1 mL). DMA (60 mg) was dissolved in ethanol (2 mL), then added to the above MNP suspension solution. Another 37 mL of Tris buffer was added to bring the total reaction volume to 40 mL. The above reaction mixture was sonicated for 2 h at room temperature under a nitrogen atmosphere, followed by overnight magnetic stirring. The MNP@DMA was collected by magnet and then washed 3 times with water and ethanol. The purified MNP@DMA was stored at −20°C until use. The amount of DMA coated on MNPs (mg DMA per mg MNPs) was quantified by measuring the difference in UV absorbance of the reaction supernatant at 480 nm by a CLARIO star plate reader (BMG Labtech, Germany) and calculated by the following equation:

(1) $$\begin{array}{c} Coated\;DMA\;per\;MNP=\frac{m\;\left(\mathrm{DMA}\;\mathrm{initial}\right)-m\;\left(\mathrm{DMA}\;\mathrm{left}\right)}{m\;\left(\mathrm{MNP}\right)}\; \end{array}$$

### 5.4 Characterization of Nanoparticles

Transmission electron microscopy (TEM, FEI electron microscope, USA) was used to image the morphology of nanoparticles. Dynamic light scattering (DLS, Malvern Zetasizer Nano, Panalytical, UK) was used to determine the hydrodynamic sizes of nanoparticles. The modification of nanoparticles was confirmed by Fourier‐transform infrared spectroscopy (FTIR, Nicolet iS50FT‐IR, Thermo Scientific, USA) with 32 scans between 400 and 4000 cm^−1^ and 0.5 cm^−1^ resolution.

### 5.5 Preparation of COPMA, COPMA‐MNP, COPMA‐MNP@DMA Hydrogels

The COPMA was synthesized according to our previous protocol by converting ∼94% of primary amines on collagen peptides to methacrylamides on COPMA (molecular weight ∼3 kg/mol [32]. A COPMA stock solution (30% w/v) was prepared in modified Dulbecco's phosphate‐buffered saline (DPBS, without calcium chloride and magnesium chloride) at room temperature and sterilized by filtering through a 0.22 µm polyethersulfone (PES) syringe filter. The solution was then diluted to 15% (w/v) COPMA by DPBS. Lithium phenyl‐2,4,6‐trimethyl‐benzoyl phosphinate (LAP) photoinitiator stock solution (4% w/v) was dissolved in DPBS, then filtered by 0.22 µm PES syringe filter. The MNP and MNP@DMA were sterilized by 70% ethanol, then dried overnight in the fumehood. The sterilized COPMA solution, at a final concentration of 15% (w/v) with 0.1% LAP, was mixed with or without MNP or MNP@DMA. The above hydrogel precursors were UV crosslinked by UV light (365 nm, 20 mW·cm^−2^) for 3 min with or without a magnetic field (35 ± 2 mT) created between 2 neodymium block magnets (46*30*10 mm, length*width*height, nickel‐plated, N40, Supermagnete, Germany).

### 5.6 Rheological Test of Hydrogels

In situ photorheological measurements were performed on a DHR‐2 rheometer (TA Instruments, USA) with a cone‐plate configuration (20 mm diameter at a 2.002° with a gap of 53 µm). Oscillatory time sweeps were conducted at 2% strain and frequency of 10 rad·s^−^^1^, initially for 1 min without UV exposure, followed by 4 min of UV irradiation (M365LP1 LED, with DC2200 LED Driver modulation, Thorlabs, Germany) at 365 nm, 20 mW·cm^−2^. Besides, oscillation frequency sweeps were performed from 1.0 to 100 rad·s^−1^ at a constant strain of 2%. Finally, oscillatory strain amplitude sweeps were carried out from 0.1% to 10% strain at 10 rad·s^−1^ frequency. Stress‐relaxation tests were conducted at 20% strain, and the stress and relaxation moduli were recorded over time.

#### 5.6.1 Self‐Healing Tests

First, strain sweep curves were recorded from 0.1%–500% strain. The cyclic strain sweeps were performed by alternatively applying 1% and 500% strain at room temperature. Besides, the deformation and reformation processes were imaged. Specifically, hydrogels were UV crosslinked in a mold (8 mm*1 mm, diameter*height) under a magnetic field (35 ± 2 mT) and UV light (UVP CL‐1000 Ultraviolet Crosslinkers, Analytik Jena, Germany) at 365 nm, 20 mW·cm^−2^ for 3 min. Finally, hydrogels were cut and reconnected at room temperature for 3 h.

### 5.7 Stability of Anisotropic Hydrogels

Anisotropic hydrogels were fabricated under a magnetic field and UV curing for 3 min as mentioned above, followed by immersing in α‐MEM (Alpha minimum essential medium with GlutaMAX, Gibco). Optical microscopy images were captured on days 0, 1, and 28 by a microscope (Eclipse Ti‐E, Nikon, Japan) to monitor degradation of the same samples over time.

### 5.8 Swelling Ratio

Hydrogels were weighed to get initial weight (W_0_), then swollen in phosphate‐buffered saline (PBS) at 37°C for 1 day and 28 days. After gently blotting hydrogels with tissue paper to remove PBS in excess, the swollen weight (W_s_) was recorded. The swelling ratio was calculated by the following equation:

(2) $$\begin{array}{c} Swelling\;ratio\;\left(\%\right)=\frac{Ws-W0}{W0}\;\times 100 \end{array}$$

### 5.9 Gel Fraction

Hydrogels were freeze dried and weighed to get initial dry weight (W_d0_), then incubated in PBS at 37°C for 1 day and 28 days. After swelling, the hydrogels were freeze‐dried again to obtain the weight (W_d1_). The gel fraction was calculated by the following equation:

(3) $$\begin{array}{c} Gel\;fraction\;\left(\%\right)=\frac{Wd1}{Wd0}\times 100 \end{array}$$

### 5.10 Cell Culture Media and Cell Culture

The proliferation medium was composed of α‐MEM supplemented with 10% (v/v) fetal bovine serum (FBS), 1% (v/v) ascorbic acid, and 1% (v/v) penicillin/streptomycin solution (Pen‐Strep, Thermo Fisher). The ligament differentiation medium was prepared fresh weekly, consisting of 4.5 g/L high‐glucose DMEM (Dulbecco's modified eagle medium, Gibco), 10% (v/v) FBS, 1% (v/v) ascorbic acid, 1% (v/v) Pen‐Strep, and 10 ng/mL TGF‐β3 (PeproTech). hMSCs from PromoCell (donor Caucasian woman, 30 years old) were cultured in T225 flasks at a density of 1000 cells/cm^2^ in proliferation medium. The cells were passaged upon reaching 80% confluence, and experiments were conducted using cells at passage 4.

### 5.11 hMSCs Laden Hydrogel

Four million/mL of hMSCs were mixed with hydrogel precursors and then UV crosslinked in a 48‐well plate under UV light (365 nm, 20 mW·cm^−2^) for 3 min with or without a magnetic field (35 ± 2 mT). Consequently, cell‐laden constructs (6.8 mm*4 mm*2 mm, length*width*height) were cultured in either proliferation or ligamentogenic differentiation media at 37°C under 5% of CO_2_ atmosphere with or without a magnetic field (35 ± 2 mT). The above media were refreshed every 2 days.

### 5.12 Live/Dead Staining

After rinsing by Dulbecco's phosphate‐buffered saline (DPBS), constructs were immersed in 2 µm of calcein acetoxymethyl solution (Calcein AM, Thermo Fisher) for 30 min at 37°C in the dark. Subsequently, ethidium homodimer‐1 solution (EthD‐1, Thermo Fisher) was added to achieve a final concentration of 0.06 µm, and the constructs were incubated for an additional 25 min at 37°C. Finally, constructs were rinsed twice with DPBS and then imaged by a fluorescence microscope (Eclipse Ti‐E, Nikon, Japan).

### 5.13 Metabolic Activity

Metabolic activity of hMSCs was quantified by Cell Titer‐Glo 3D assay (Promega) on days 0, 1, 7, 14, and 28. Specifically, equal volumes of reagent and medium were added into the hMSC‐laden hydrogels and then mixed by pipetting. After 30 min incubation at room temperature, 100 µL of supernatant was transferred to a white bottom 96 well plate and luminescence was measured by a CLARIO star plate reader (BMG Labtech, Germany).

### 5.14 DNA Quantification

Cell extraction from hMSC‐laden hydrogels was obtained by freeze‐thawing 3 times, digesting overnight at 56°C with 1 mg/mL proteinase K, and freeze‐thawing 3 more times. DNA quantification was performed following the protocol provided by the CyQuant cell proliferation assay kit (Thermo Fisher, USA). Briefly, the supernatant from the above cell extraction was further incubated with lysis buffer containing RNase for 2 h at room temperature. An equal volume of 2× GR‐dye solution was added to the mixture followed by incubation for 15 min. Samples (200 µL) were transferred to a black 96‐well plate, and the fluorescence at 520 nm was measured by a CLARIO star plate reader.

### 5.15 GAG Quantification

Cell extraction (25 µL), calcium chloride (5 µL), and 1,9‐dimethylmethylene blue solution (150 µL) were mixed in a 96‐well plate. The absorbance was measured at 525 nm and 595 nm by a CLARIO star plate reader, and the difference between these readings was used for quantification.

### 5.16 Immunostaining of YAP, COL‐I, and SCX

Constructs were fixed in 4% paraformaldehyde (PFA) and then rinsed with PBS. Constructs were blocked and permeabilized overnight at 4°C with 1% (w/v) Triton X‐100, 0.05% (w/v) Tween‐20, 5% (w/v) goat serum, and 1% (w/v) Bovine Serum Albumin (BSA, VWR) in PBS. After removing the above solution, constructs were incubated with rabbit anti‐YAP (YAP, 1:200, ab52771, Abcam) or mouse anti‐collagen I (COL‐I, 1:200, ab6308, Abcam) and rabbit anti‐Scleraxis (SCX, 1:200, ab58655, Abcam) primary antibodies. After incubation for 24 h at 4°C, primary antibodies were washed with washing buffer consisting of 1% BSA and 0.05% Tween‐20 in PBS. Constructs were incubated overnight at 4°C with secondary antibodies (goat anti‐mouse and goat anti‐rabbit, both at 1:500 in washing buffer, Abcam). After washing the secondary antibody, F‐actin was stained by Alexa Fluor Phalloidin 568 (1:75, Thermo Fisher) in PBS for 1 h, followed by DNA staining with DAPI for 20 min in the dark at room temperature. After rinsing with PBS, samples were imaged with a confocal microscope (Leica TCS SP8 CARS, Germany). The quantification of angle distribution of MNP chains and cells, nucleus aspect ratio, cell number, F‐actin area, YAP_nucleus/cytoplasm_, COL‐I, and SCX expression were calculated by NIS‐Elements software with general analysis (GA3) module (Nikon, version 5.30.3, Japan).

### 5.17 Statistical Analysis

Statistical analysis was performed using GraphPad Prism software (version 10.3, USA). Student's *t*‐test, one‐way and two‐way ANOVA with Tukey's multiple comparison test were used to assess statistical significance. Data are expressed as mean ± standard deviation (SD) or shown with SD bars in the graphs. Values were considered significant when ^*^ *p* < 0.05; ^**^ *p* < 0.01; ^***^ *p* < 0.001 and ^****^ *p* < 0.0001.

## Conflicts of Interest

The authors declare no conflicts of interest.

## Supporting information




**Supporting File**: smll73663‐sup‐0001‐SuppMat.pdf.

## Data Availability

The data that support the findings of this study are available from the corresponding author upon reasonable request.

## References

[1] M. Mao , K. Han , J. Gao , et al., “Engineering Highly Aligned and Densely Populated Cardiac Muscle Bundles via Fibrin Remodeling in 3D‐Printed Anisotropic Microfibrous Lattices,” Advanced Materials 37 (2025): 2419380, 10.1002/adma.202419380. 39811972

[2] J. Xing , N. Liu , N. Xu , W. Chen , and D. Xing , “Engineering Complex Anisotropic Scaffolds beyond Simply Uniaxial Alignment for Tissue Engineering,” Advanced Functional Materials 32, no. 15 (2021): 2110676, 10.1002/adfm.202110676.

[3] A. Yaguchi , M. Oshikawa , G. Watanabe , et al., “Efficient Protein Incorporation and Release by a Jigsaw‐Shaped Self‐Assembling Peptide Hydrogel for Injured Brain Regeneration,” Nature Communications 12, no. 1 (2021): 6623, 10.1038/s41467-021-26896-3. PMC8604910 34799548

[4] B. G. Soliman , A. K. Nguyen , J. J. Gooding , and K. A. Kilian , “Advancing Synthetic Hydrogels through Nature‐Inspired Materials Chemistry,” Advanced Materials 36, no. 42 (2024): 2404235, 10.1002/adma.202404235. PMC11486603 38896849

[5] X. Wan , Z. Liu , and L. Li , “Manipulation of Stem Cells Fates: the Master and Multifaceted Roles of Biophysical Cues of Biomaterials,” Advanced Functional Materials 31, no. 23 (2021): 2010626, 10.1002/adfm.202010626.

[6] M. Volpi , A. Paradiso , M. Costantini , and W. Swieszkowski , “Hydrogel‐Based fiber Biofabrication Techniques for Skeletal Muscle Tissue Engineering,” ACS Biomaterials Science & Engineering 8, no. 2 (2022): 379–405, 10.1021/acsbiomaterials.1c01145. 35084836 PMC8848287

[7] W. Kitana , I. Apsite , and L. Ionov , “3d (bio) printing Combined fiber Fabrication Methods for Tissue Engineering Applications: Possibilities and Limitations,” Advanced Functional Materials (2025): 2500450, 10.1002/adfm.202500450.

[8] K. Yao , S. Lv , X. Zhang , et al., “3d printing of Multiscale Biomimetic Scaffold for Tendon Regeneration,” Advanced Functional Materials 35, no. 4 (2024): 2413970, 10.1002/adfm.202413970.

[9] R. P. Rimington , A. J. Capel , S. D. R. Christie , and M. P. Lewis , “Biocompatible 3d Printed Polymers via Fused Deposition Modelling Direct c_2_c_12_ Cellular Phenotype in Vitro,” Lab on a Chip 17, no. 17 (2017): 2982–2993, 10.1039/C7LC00577F. 28762415

[10] L. Sun , Y. Wang , F. Bian , D. Xu , and Y. Zhao , “Bioinspired Optical and Electrical Dual‐Responsive Heart‐on‐a‐Chip for Hormone Testing,” Science Bulletin 68, no. 9 (2023): 938–945, 10.1016/j.scib.2023.04.010. 37062651

[11] W. Wang , A. Wang , G. Hu , et al., “Potential of an Aligned Porous Hydrogel Scaffold Combined with Periodontal Ligament Stem Cells or Gingival Mesenchymal Stem Cells to Promote Tissue Regeneration in Rat Periodontal Defects,” ACS Biomaterials Science & Engineering 9, no. 4 (2023): 1961–1975, 10.1021/acsbiomaterials.2c01440. 36942823

[12] M. T. I. Mredha , Y. Z. Guo , T. Nonoyama , T. Nakajima , T. Kurokawa , and J. P. Gong , “A Facile Method to Fabricate Anisotropic Hydrogels with Perfectly Aligned Hierarchical Fibrous Structures,” Advanced Materials 30, no. 9 (2018): 1704937. 10.1002/adma.201704937 29341264

[13] G. Cedillo‐Servin , E. A. A. Al‐Jehani , T. Rossy , et al., “Meta‐Adaptive Biomaterials: Multiscale, Spatiotemporal Organization and Actuation in Engineered Tissues,” Trends in Biotechnology 43 (2025): 2709–2723. 40473558 10.1016/j.tibtech.2025.05.004

[14] J. Li , G. Li , X. Lu , et al., “Magnetically Responsive Optical Modulation: from Anisotropic Nanostructures to Emerging Applications,” Advanced Functional Materials 34, no. 3 (2023): 2308293.

[15] N. Demri , S. Dumas , M. L. Nguyen , et al., “Remote Magnetic Microengineering and Alignment of Spheroids into 3d Cellular Fibers,” Advanced Functional Materials 32, no. 50 (2022): 2204850, 10.1002/adfm.202204850.

[16] A. Pardo , S. M. Bakht , M. Gomez‐Florit , et al., “Magnetically‐Assisted 3d Bioprinting of Anisotropic Tissue‐Mimetic Constructs,” Advanced Functional Materials 32, no. 50 (2022): 2208940.

[17] A. Tokarev , O. Trotsenko , I. M. Griffiths , H. A. Stone , and S. Minko , “Magnetospinning of Nano‐ and Microfibers,” Advanced Materials 27, no. 23 (2015): 3560–3565, 10.1002/adma.201500374. 25953082

[18] R. K. Tindell , L. P. Busselle , and J. L. Holloway , “Magnetic Fields Enable Precise Spatial Control over Electrospun fiber Alignment for Fabricating Complex Gradient Materials,” Journal of Biomedical Materials Research Part A 111, no. 6 (2023): 778–789, 10.1002/jbm.a.37492. 36594559

[19] D. L. Braunmiller , S. Babu , D. B. Gehlen , et al., “Pre‐Programmed Rod‐Shaped Microgels to Create Multi‐Directional Anisogels for 3D Tissue Engineering,” Advanced Functional Materials 32, no. 50 (2022): 2202430, 10.1002/adfm.202202430.

[20] B. R. Nelson , B. E. Kirkpatrick , C. E. Miksch , et al., “Photoinduced Dithiolane Crosslinking for Multiresponsive Dynamic Hydrogels,” Advanced Materials 36, no. 43 (2024): 2211209, 10.1002/adma.202211209. PMC10387131 36715698

[21] B. A. Nerger , K. Kashyap , B. T. Deveney , et al., “Tuning Porosity of Macroporous Hydrogels Enables Rapid Rates of Stress Relaxation and Promotes Cell Expansion and Migration,” Proceedings of the National Academy of Sciences 121, no. 45 (2024): 2410806121, 10.1073/pnas.2410806121. PMC11551365 39467139

[22] O. Chaudhuri , L. Gu , D. Klumpers , et al., “Hydrogels with Tunable Stress Relaxation Regulate Stem Cell Fate and Activity,” Nature Materials 15, no. 3 (2016): 326–334, 10.1038/nmat4489. 26618884 PMC4767627

[23] Y. Zhang , M. Remy , T. Leste‐Lasserre , and M. C. Durrieu , “Manipulating Stem Cell Fate with Disordered Bioactive Cues on Surfaces: the Role of Bioactive Ligand Selection,” ACS Applied Materials & Interfaces 16, no. 15 (2024): 18474–18489, 10.1021/acsami.4c00262. 38581548

[24] P. Seth , J. Friedrichs , Y. D. P. Limasale , et al., “Interpenetrating Polymer Network Hydrogels with Tunable Viscoelasticity and Proteolytic Cleavability to Direct Stem Cells in Vitro,” Advanced Healthcare Materials 14, no. 9 (2025): 2402656, 10.1002/adhm.202402656. 39506429 PMC11973941

[25] L. Zhao , Y. Lai , H. Jiao , and J. Huang , “Nerve Growth Factor Receptor Limits Inflammation to Promote Remodeling and Repair of Osteoarthritic Joints,” Nature Communications 15, no. 1 (2024): 3225, 10.1038/s41467-024-47633-6. PMC11018862 38622181

[26] I. Donderwinkel , R. S. Tuan , N. R. Cameron , and J. E. Frith , “A Systematic Investigation of the Effects of TGF‐β3 and Mechanical Stimulation on Tenogenic Differentiation of Mesenchymal Stromal Cells in a Poly(ethylene glycol)/Gelatin‐Based Hydrogel,” Journal of Orthopaedic Translation 43 (2023): 1–13, 10.1016/j.jot.2023.09.006. 37929240 PMC10622696

[27] I. Grafe , S. Alexander , J. R. Peterson , et al., “TGF‐β Family Signaling in Mesenchymal Differentiation,” Cold Spring Harbor Perspectives in Biology 10, no. 5 (2018): a022202, 10.1101/cshperspect.a022202. 28507020 PMC5932590

[28] H. Nosrati , M. Salehiabar , M. Fridoni , et al., “New Insight about Biocompatibility and Biodegradability of Iron Oxide Magnetic Nanoparticles: Stereological and in Vivo Mri Monitor,” Scientific Reports 9, no. 1 (2019): 7173. 31073222 10.1038/s41598-019-43650-4 PMC6509211

[29] A. Fromain , J. E. Perez , A. Van de Walle , Y. Lalatonne , and C. Wilhelm , “Photothermia at the Nanoscale Induces Ferroptosis via Nanoparticle Degradation,” Nature Communications 14, no. 1 (2023): 4637, 10.1038/s41467-023-40258-1. PMC10397343 37532698

[30] A. R. Abdel Fattah , N. Kolaitis , K. Van Daele , B. Daza , A. G. Rustandi , and A. Ranga , “Targeted Mechanical Stimulation via Magnetic Nanoparticles Guides in Vitro Tissue Development,” Nature Communications 14, no. 1 (2023): 5281, 10.1038/s41467-023-41037-8. PMC10465512 37644160

[31] B. Rezaei , P. Yari , S. M. Sanders , et al., “Magnetic Nanoparticles: a Review on Synthesis, Characterization, Functionalization, and Biomedical Applications,” Small 20, no. 5 (2024): 2304848, 10.1002/smll.202304848. 37732364

[32] H. Weng , M. C. Decarli , L. He , et al., “Mechanical Reinforced and Self‐Healing Hydrogels: Bioprinted Biomimetic Methacrylated Collagen Peptide‐Xanthan Gum Constructs for Ligament Regeneration,” Advanced Healthcare Materials 14, no. 25 (2025): 2502341, 10.1002/adhm.202502341. 40665850 PMC12477574

[33] W. Chen , Y. Zhang , J. Kumari , H. Engelkamp , and P. H. J. Kouwer , “Magnetic Stiffening in 3d Cell Culture Matrices,” Nano Letters 21, no. 16 (2021): 6740–6747, 10.1021/acs.nanolett.1c00371. 34387494 PMC8392345

[34] A. Zengin , J. P. O. Castro , P. Habibovic , and S. H. van Rijt , “Injectable, Self‐Healing Mesoporous Silica Nanocomposite Hydrogels with Improved Mechanical Properties,” Nanoscale 13, no. 2 (2021): 1144–1154, 10.1039/D0NR07406C. 33400753 PMC8100892

[35] N. Liu , J. Jiang , T. Liu , H. Chen , and N. Jiang , “Compositional, Structural, and Biomechanical Properties of Three Different Soft Tissue–Hard Tissue Insertions: a Comparative Review,” ACS Biomaterials Science & Engineering 10, no. 5 (2024): 2659–2679, 10.1021/acsbiomaterials.3c01796. 38697939

[36] W. Chen , J. Kumari , H. Yuan , F. Yang , and P. H. J. Kouwer , “Toward Tissue‐like Material Properties: Inducing in Situ Adaptive Behavior in Fibrous Hydrogels,” Advanced Materials 34, no. 37 (2022): 2202057, 10.1002/adma.202202057. 35792703

[37] X. Qi , E. Cai , Y. Xiang , et al., “An Immunomodulatory Hydrogel by Hyperthermia‐Assisted Self‐Cascade Glucose Depletion and ROS Scavenging for Diabetic Foot Ulcer Wound Therapeutics,” Advanced Materials 35, no. 48 (2023): 2306632, 10.1002/adma.202306632. 37803944

[38] Z. Wang , G. An , Y. Zhu , et al., “3D‐Printable Self‐Healing and Mechanically Reinforced Hydrogels with Host–Guest Non‐Covalent Interactions Integrated into Covalently Linked Networks,” Materials Horizons 6, no. 4 (2019): 733–742, 10.1039/C8MH01208C. 31572613 PMC6768557

[39] Y. J. No , M. Castilho , Y. Ramaswamy , and H. Zreiqat , “Role of Biomaterials and Controlled Architecture on Tendon/Ligament Repair and Regeneration,” Advanced Materials 32, no. 18 (2020): 1904511, 10.1002/adma.201904511. 31814177

[40] N. L. Leong , J. L. Kator , T. L. Clemens , A. James , M. Enamoto‐Iwamoto , and J. Jiang , “Tendon and Ligament Healing and Current Approaches to Tendon and Ligament Regeneration,” Journal of Orthopaedic Research 38, no. 1 (2020): 7–12, 10.1002/jor.24475. 31529731 PMC7307866

[41] S. Dupont , L. Morsut , M. Aragona , et al., “Role of yap/taz in Mechanotransduction,” Nature 474, no. 7350 (2011): 179–183, 10.1038/nature10137. 21654799

[42] R. Nakamichi and H. Asahara , “Regulation of Tendon and Ligament Differentiation,” Bone 143 (2021): 115609, 10.1016/j.bone.2020.115609. 32829041 PMC7770025

[43] F. Atarbashi‐Moghadam , A. Azadi , H. Nokhbatolfoghahaei , and N. Taghipour , “Effect of Simultaneous and Sequential Use of TGF‐β1 and TGF‐β3 with FGF‐2 on Teno/Ligamentogenic Differentiation of Periodontal Ligament Stem Cells,” Archives of Oral Biology 162 (2024): 105956, 10.1016/j.archoralbio.2024.105956. 38522213

[44] C. Xiao , N. Xie , Q. Shu , et al., “Synergistic Effects of Matrix Biophysical Properties on Gastric Cancer Cell Behavior via Integrin‐Mediated Cell‐ECM Interactions,” Small 20, no. 36 (2024): 2309907, 10.1002/smll.202309907. 38712486

[45] Y. Ma , T. Han , Q. Yang , et al., “Viscoelastic Cell Microenvironment: Hydrogel‐Based Strategy for Recapitulating Dynamic ECM Mechanics,” Advanced Functional Materials 31, no. 24 (2021): 2100848, 10.1002/adfm.202100848.

[46] W. J. Han , J. H. Lee , J.‐K. Lee , and H. J. Choi , “Remote‐Controllable, Tough, Ultrastretchable, and Magneto‐Sensitive Nanocomposite Hydrogels with Homogeneous Nanoparticle Dispersion as Biomedical Actuators, and Their Tuned Structure, Properties, and Performances,” Composites Part B: Engineering 236 (2022): 109802, 10.1016/j.compositesb.2022.109802.

[47] P. V. Londhe , M. V. Londhe , A. B. Salunkhe , et al., “Magnetic Hydrogel (maggel): an Evolutionary Pedestal for Anticancer Therapy,” Coordination Chemistry Reviews 522 (2025): 216228, 10.1016/j.ccr.2024.216228.

[48] E. V. Araujo , S. V. Carneiro , D. M. A. Neto , et al., “Advances in Surface Design and Biomedical Applications of Magnetic Nanoparticles,” Advances in Colloid and Interface Science 328 (2024): 103166, 10.1016/j.cis.2024.103166. 38728773

[49] S. Richard , A. K. A. Silva , G. Mary , et al., “3d magnetic Alignment of Cardiac Cells in Hydrogels,” ACS Applied Bio Materials 3, no. 10 (2020): 6802–6810, 10.1021/acsabm.0c00754. 35019343

[50] M. S. Islam , T. G. Molley , T. T. Hung , et al., “Magnetic Nanofibrous Hydrogels for Dynamic Control of Stem Cell Differentiation,” ACS Applied Materials & Interfaces 15, no. 44 (2023): 50663–50678, 10.1021/acsami.3c07021. 37643902

[51] M. S. Islam , T. G. Molley , G. K. Jalandhra , J. Fang , J. J. Kruzic , and K. A. Kilian , “Magnetoactive Nanotopography on Hydrogels for Stimulated Cell Adhesion and Differentiation,” Small Science 5, no. 4 (2025): 2400468, 10.1002/smsc.202400468. 40657193 PMC12244508

[52] M. S. Islam , T. G. Molley , J. Ireland , J. J. Kruzic , and K. A. Kilian , “Magnetic Nanocomposite Hydrogels for Directing Myofibroblast Activity in Adipose‐Derived Stem Cells,” Advanced NanoBiomed Research 1, no. 4 (2021): 2000072, 10.1002/anbr.202000072.

[53] Y. Xu , H. Yin , J. Chu , D. Eglin , T. Serra , and D. Docheva , “An Anisotropic Nanocomposite Hydrogel Guides Aligned Orientation and Enhances Tenogenesis of human Tendon Stem/Progenitor Cells,” Biomaterials Science 9, no. 4 (2021): 1237–1245, 10.1039/D0BM01127D. 33576754

[54] Y. Ma , A. Ma , T. Luo , S. Xiao , and H. Zhou , “Fabrication of Anisotropic Nanocomposite Hydrogels by Magnetic Field‐Induced Orientation for Mimicking Cardiac Tissue,” Journal of Applied Polymer Science 140, no. 1 (2022): 53248, 10.1002/app.53248.

[55] M. Shi , L. Bai , M. Xu , et al., “Magnetically Induced Anisotropic Conductive in Situ Hydrogel for Skeletal Muscle Regeneration by Promoting Cell Alignment and Myogenic Differentiation,” Chemical Engineering Journal 484 (2024): 149019, 10.1016/j.cej.2024.149019.

[56] A. L. Wright , L. Righelli , T. J. Broomhall , H. C. Lamont , and A. J. El Haj , “Magnetic Nanoparticle‐Mediated Orientation of Collagen Hydrogels for Engineering of Tendon‐Mimetic Constructs,” Frontiers in Bioengineering and Biotechnology 10 (2022): 797437, 10.3389/fbioe.2022.797437. 35372293 PMC8968910

[57] T. U. Nguyen , K. E. Watkins , and V. Kishore , “Photochemically Crosslinked Cell‐Laden Methacrylated Collagen Hydrogels with High Cell Viability and Functionality,” Journal of Biomedical Materials Research Part A 107, no. 7 (2019): 1541–1550, 10.1002/jbm.a.36668. 30882990 PMC6527486

[58] D. Bennet and S. Kim , “Evaluation of Uv Radiation‐Induced Toxicity and Biophysical Changes in Various Skin Cells with Photo‐Shielding Molecules,” The Analyst 140, no. 18 (2015): 6343–6353, 10.1039/C5AN00979K. 26247629

[59] C. Naud , C. Thebault , M. Carriere , et al., “Cancer Treatment by Magneto‐Mechanical Effect of Particles, a Review,” Nanoscale Advances 2, no. 9 (2020): 3632–3655, 10.1039/D0NA00187B. 36132753 PMC9419242

[60] M. Domenech , I. Marrero‐Berrios , M. Torres‐Lugo , and C. Rinaldi , “Lysosomal Membrane Permeabilization by Targeted Magnetic Nanoparticles in Alternating Magnetic Fields,” ACS Nano 7, no. 6 (2013): 5091–5101, 10.1021/nn4007048. 23705969

[61] T. Kuhnt , S. Camarero‐Espinosa , M. Takhsha Ghahfarokhi , et al., “4d printed Shape Morphing Biocompatible Materials Based on Anisotropic Ferromagnetic Nanoparticles,” Advanced Functional Materials 32, no. 50 (2022): 2202539.

[62] J. Shorrocks , N. D. Paul , and T. J. McMillan , “The Dose Rate of Uva Treatment Influences the Cellular Response of Hacat Keratinocytes,” Journal of Investigative Dermatology 128, no. 3 (2008): 685–693, 10.1038/sj.jid.5701037. 17762856

[63] D. E. Godar , C. Gurunathan , and I. Ilev , “3d bioprinting with uva1 Radiation and Photoinitiator irgacure 2959: Can the astm Standard l929 Cells Predict human Stem Cell Cytotoxicity?,” Photochemistry and Photobiology 95, no. 2 (2019): 581–586, 10.1111/php.13028. 30267574

[64] T. J. McMillan , E. Leatherman , A. Ridley , J. Shorrocks , S. E. Tobi , and J. R. Whiteside , “Cellular Effects of Long Wavelength Uv Light (uva) in Mammalian Cells,” Journal of Pharmacy and Pharmacology 60, no. 8 (2008): 969–976, 10.1211/jpp.60.8.0004. 18644190

[65] C. Thebault , M. Marmiesse , C. Naud , et al., “Magneto‐Mechanical Treatment of human Glioblastoma Cells with Engineered Iron Oxide Powder Microparticles for Triggering Apoptosis,” Nanoscale Advances 3, no. 21 (2021): 6213–6222, 10.1039/D1NA00461A. 36133951 PMC9418695

[66] C. Choi , E. Yun , M. Song , J. Kim , J. S. Son , and C. Cha , “Multiscale Control of Nanofiber‐Composite Hydrogel for Complex 3d Cell Culture by Extracellular Matrix Composition and Nanofiber Alignment,” Biomaterials Research 28 (2024): 0032, 10.34133/bmr.0032. 38812742 PMC11136538

[67] J. C. Rose , M. Camara‐Torres , K. Rahimi , J. Kohler , M. Moller , and L. De Laporte , “Nerve Cells Decide to Orient inside an Injectable Hydrogel with Minimal Structural Guidance,” Nano Letters 17, no. 6 (2017): 3782–3791, 10.1021/acs.nanolett.7b01123. 28326790 PMC5537692

[68] J. Nowak‐Jary and B. Machnicka , “Toxicity of Magnetic Nanoparticles in Medicine: Contributing Factors and Modern Assessment Methods,” International Journal of Molecular Sciences 26, no. 17 (2025): 8586, 10.3390/ijms26178586. 40943506 PMC12428864

[69] S. Shalom , E. Kuznetsova , G. Shklarski Shchori , et al., “The Influence of Magnetothermal Stimulation on Viability of Cells in 2d Cultures and 3d Magnetic Collagen Gels,” Advanced Electronic Materials 11, no. 17 (2025), 10.1002/aelm.202500105.

[70] Y. Ma , J. Yang , Y. Hu , Z. Xia , and K. Cai , “Osteogenic Differentiation of the MSCs on Silk Fibroin Hydrogel Loaded Fe_3_O_4_@PAA NPs in Static Magnetic Field Environment,” Colloids and Surfaces B: Biointerfaces 220 (2022): 112947, 10.1016/j.colsurfb.2022.112947. 36272283

[71] A. Vinhas , M. T. Rodrigues , A. I. Goncalves , R. L. Reis , and M. E. Gomes , “Magnetic Responsive Materials Modulate the Inflammatory Profile of IL‐1β Conditioned Tendon Cells,” Acta Biomaterialia 117 (2020): 235–245, 10.1016/j.actbio.2020.09.028. 32966921

[72] E. Qiao , J. Baek , C. Fulmore , et al., “Spectrin Mediates 3d‐Specific Matrix Stress‐Relaxation Response in Neural Stem Cell Lineage Commitment,” Science Advances 10, no. 31 (2024): adk8232, 10.1126/sciadv.adk8232. PMC11296331 39093963

[73] B. A. Nerger , S. Sinha , N. N. Lee , et al., “3d hydrogel Encapsulation Regulates Nephrogenesis in Kidney Organoids,” Advanced Materials 36, no. 14 (2024): 2308325, 10.1002/adma.202308325. PMC10994733 38180232

[74] A. Elosegui‐Artola , A. Gupta , A. J. Najibi , et al., “Matrix Viscoelasticity Controls Spatiotemporal Tissue Organization,” Nature Materials 22, no. 1 (2023): 117. 36456871 10.1038/s41563-022-01400-4 PMC10332325

[75] S. Babu , I. Chen , S. Vedaraman , et al., “How Do the Local Physical, Biochemical, and Mechanical Properties of an Injectable Synthetic Anisotropic Hydrogel Affect Oriented Nerve Growth?,” Advanced Functional Materials 32, no. 50 (2022): 2202468, 10.1002/adfm.202202468.

