# Supplementary material for: Dual‐Responsive Hydrogels Engineer Anisotropic Cellular Microenvironment to Modulate Stem Cell Organization and Fate
Source: Small. 2026 May 14;22(36):e00072. doi: 10.1002/smll.202600072 (PMC13307265; doi:10.1002/smll.202600072)
Supplement: Supplementary file 1 — Supporting File: smll73663‐sup‐0001‐SuppMat.pdf. [file SMLL-22-e00072-s001.pdf]

Supporting information

Dual-Responsive Hydrogels Engineer Anisotropic Cellular Microenvironment to Modulate Stem Cell Organization and Fate

Hongjuan Weng[1,2], Wen Chen[1], Lei He[3], Timo Rademakers[1], Sabine van Rijt[3], Monize C. Decarli[1,4], Katrien V. Bernaerts[2], Lorenzo Moroni[1*]

1 Complex Tissue Regeneration Department, MERLN Institute for Technology Inspired Regenerative Medicine, Maastricht University, The Netherlands

2 Sustainable Polymer Synthesis Group, Aachen-Maastricht Institute for Biobased Materials, Maastricht University, The Netherlands

3 Instructive Biomaterials Engineering Department, MERLN Institute for Technology-Inspired Regenerative Medicine, Maastricht University, The Netherlands

4 Department of Biomaterials and Biomedical Technology, University Medical Center Groningen, University of Groningen, The Netherlands

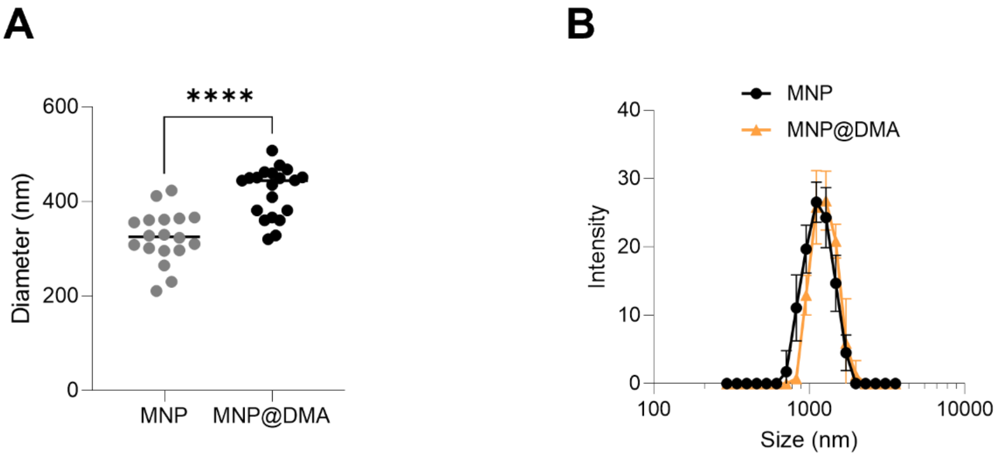

Figure S1. Characterization of MNP and MNP@DMA. A) Diameter analyzed from TEM (n ≥ 18, ****p < 0.0001). B) Hydrodynamic diameter obtained from DLS (n = 3).

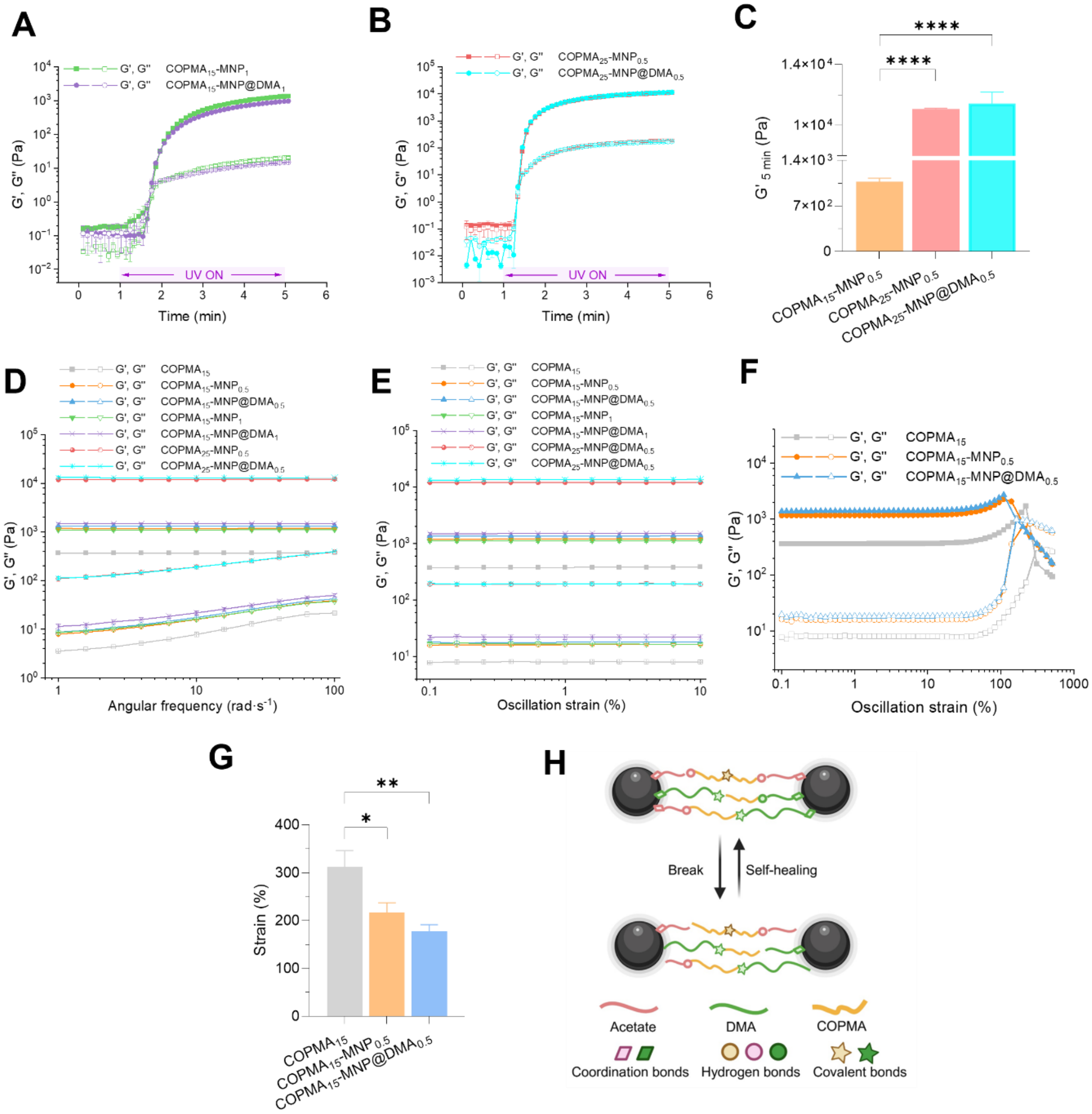

Figure S2. A) Rheological measurements of $COPMA_{15}$-MNP_1, $COPMA_{15}$-$MNP@DMA_1$ hydrogels. B) Rheological measurements of $COPMA_{25}$-$MNP_{0.5}$, and $COPMA_{25}$-$MNP@DMA_{0.5}$ hydrogels. C) Storage modulus at 5 min ($G'_{5\ min}$) of $COPMA_{15}$-$MNP_{0.5}$, $COPMA_{25}$-$MNP_{0.5}$, and $COPMA_{25}$-$MNP@DMA_{0.5}$ hydrogels. D) Frequency sweep from 1 to 100 $rad \cdot s^{-1}$, with a constant strain of 2%. E) Strain sweep mode from 1 to 10% with a constant frequency of 10 $rad \cdot s^{-1}$. F) Strain sweeps of $COPMA_{15}$, $COPMA_{15}$-$MNP_{0.5}$, and $COPMA_{15}$-$MNP@DMA_{0.5}$ hydrogels from 0.1% to 500% at an angular frequency of 10 $rad \cdot s^{-1}$. G) Yield strain of $COPMA_{15}$, $COPMA_{15}$-$MNP_{0.5}$, and $COPMA_{15}$-$MNP@DMA_{0.5}$ hydrogels under strain sweep mode from 1 to 500% with a constant frequency of 10 $rad \cdot s^{-1}$. Data were averaged from experiments repeated at least two times, and error bars represent standard deviation. H) Mechanism of self-healing hydrogels.

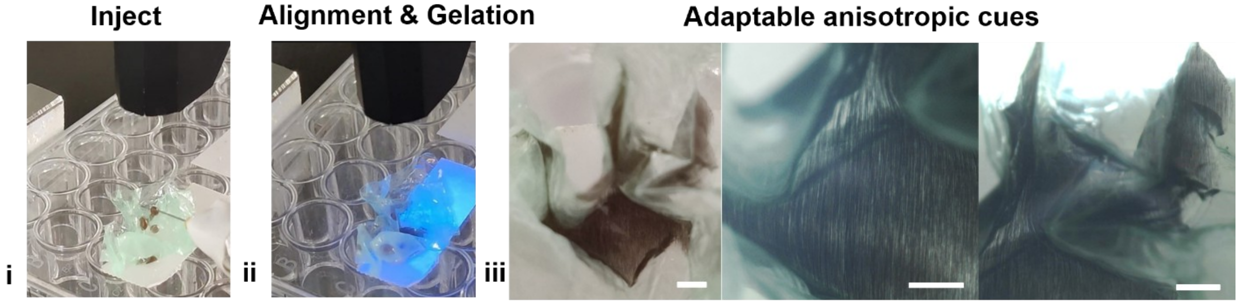

Figure S3. Images of nanocomposite hydrogels with magnetic-light-responsive properties and adaptable anisotropic morphology. i) Hydrogel precursor was injected into an irregular shape container. ii) Once the injection was finished, UV light was turned on in the presence of magnetic field. iii) The anisotropic cues were adaptable to the irregular shape container. Scale bar: 200 µm.

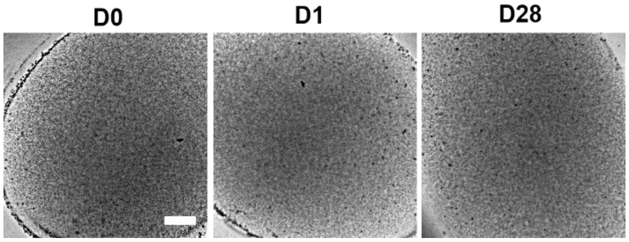

Figure S4. Optical images of $COPMA_{15}$-$MNP_{0.5}$ hydrogels without magnetic field, scale bar: 500 µm.

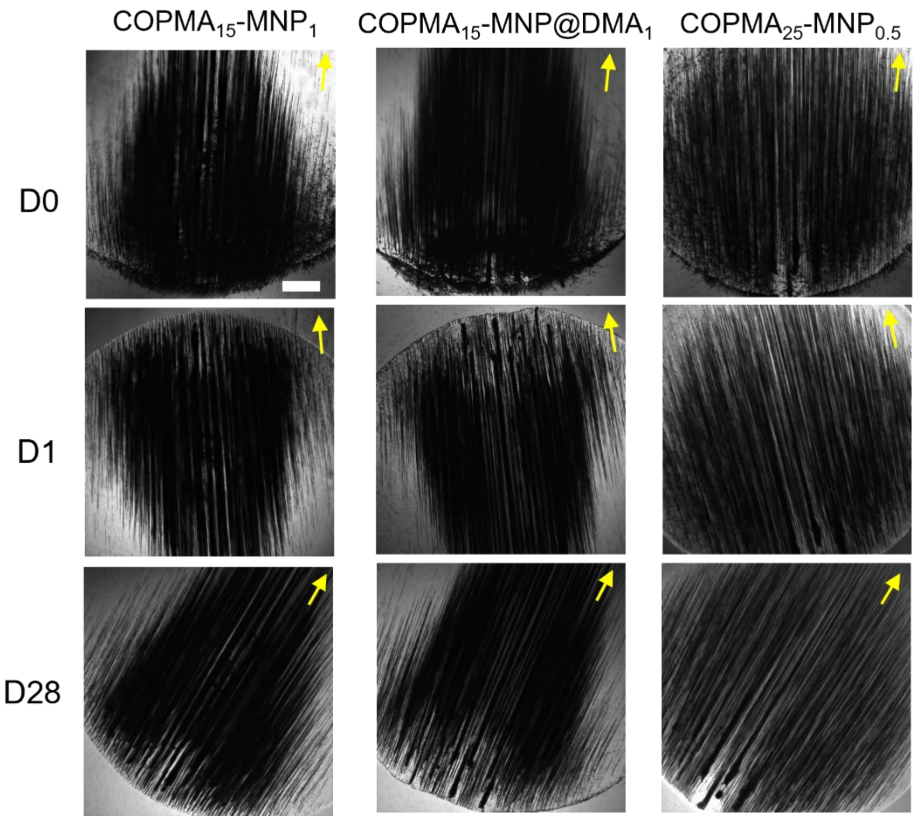

Figure S5. Optical images of $COPMA_{15}$-MNP_1, $COPMA_{15}$-$MNP@DMA_1$ and $COPMA_{25}$-$MNP_{0.5}$ hydrogels, scale bar: 500 μm. Yellow arrows indicate the direction of the magnetic field.

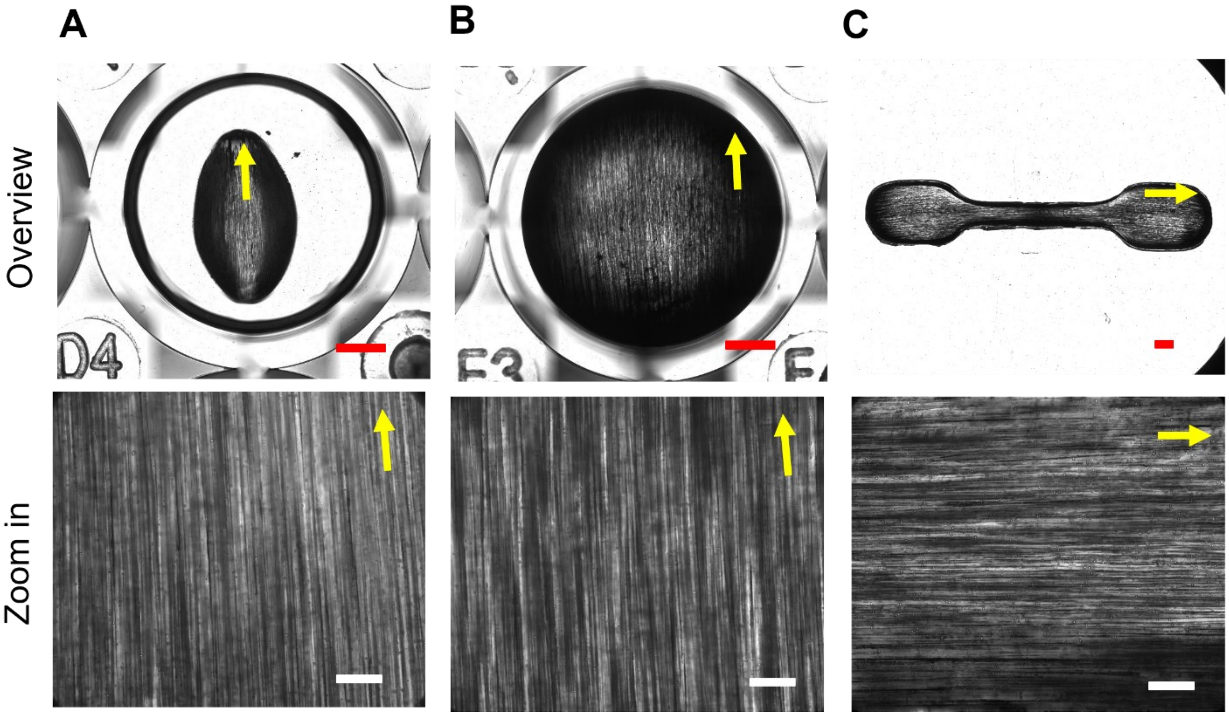

Figure S6. Optical images of magnetic nanocomposite hydrogels in various sizes. A) Droplet size: 6.8 mm*4 mm*2 mm, length*width*height. B) Cilinder size: 10.4 mm*2 mm, diameter*height. C) Strip size: 39 mm*7.5 mm*2 mm, length*width*height. Red scale bar: 2000 μm. White scale bar: 200 μm. Yellow arrows indicate the direction of the magnetic field.

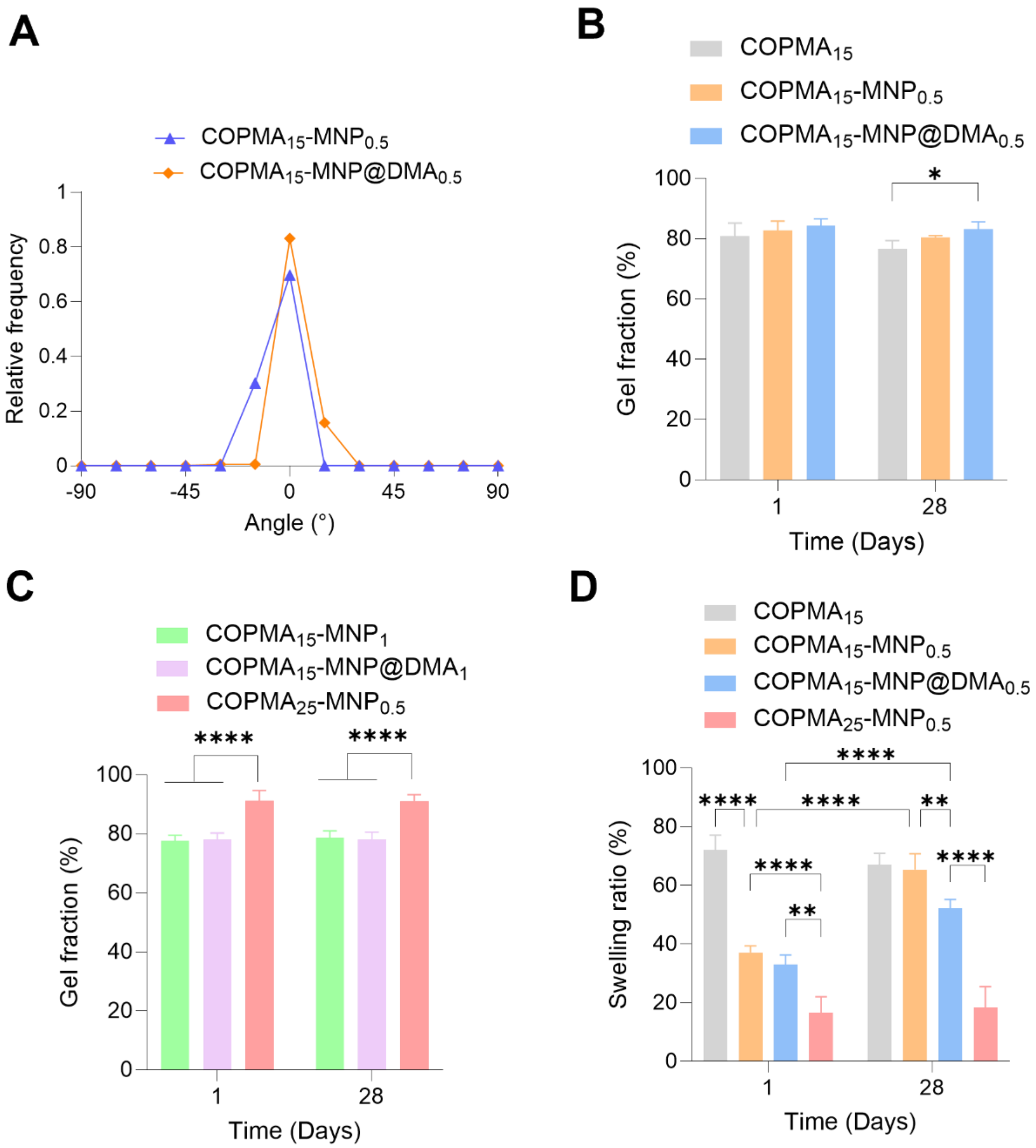

Figure S7. A) Angle distribution of nanoparticle chains in $COPMA_{15}$-$MNP_{0.5}$, and $COPMA_{15}$-MNP@$DMA_{0.5}$ hydrogels with magnetic field. B-C) Gel fraction of hydrogels ($n \geq 3$). Due to tighter hydrogel networks by more noncovalent bonds or covalent bonds, gel fraction of nanocomposite hydrogels was higher than that of $COPMA_{15}$ hydrogels on day 1 and 28. Especially on day 28, the gel fraction of $COPMA_{15}$-MNP@$DMA_{0.5}$ hydrogel (83.1% ± 2.3%) was significantly higher than that of $COPMA_{15}$ hydrogels (76.6% ± 2.4%). The $COPMA_{25}$-$MNP_{0.5}$ hydrogel showed higher gel fraction (91.3% ± 2.9%) at day 1 and remained stable over 28 days. Thus, the gel fraction of COPMA hydrogels could be improved by adding nanoparticles, which could be further increased by increasing the concentration of COPMA. D) Swelling ratio of hydrogels ($n \geq 3$, *$p < 0.05$, **$p < 0.01$, ****$p < 0.0001$). Swelling ratios of $COPMA_{15}$-$MNP_{0.5}$ (37.0 % ± 2.1%) and $COPMA_{15}$-MNP@$DMA_{0.5}$ (33.1% ± 2.8%) hydrogels were significantly lower than that of $COPMA_{15}$ hydrogels (72.0% ± 4.6%) on day 1. On day 28, swelling ratios of

$COPMA_{15}$-$MNP_{0.5}$ and $COPMA_{15}$-MNP@$DMA_{0.5}$ hydrogels were significantly increased to 65.3% ± 5.0% and 52.2% ± 2.7%, indicating their tighter swelling network than $COPMA_{15}$ hydrogel (Figure S6D). The swelling ratio of $COPMA_{25}$-$MNP_{0.5}$ was significantly lower than that of $COPMA_{15}$-$MNP_{0.5}$ hydrogel on day 1 and 28, indicating more condensed hydrogel network in higher COPMA concentration.

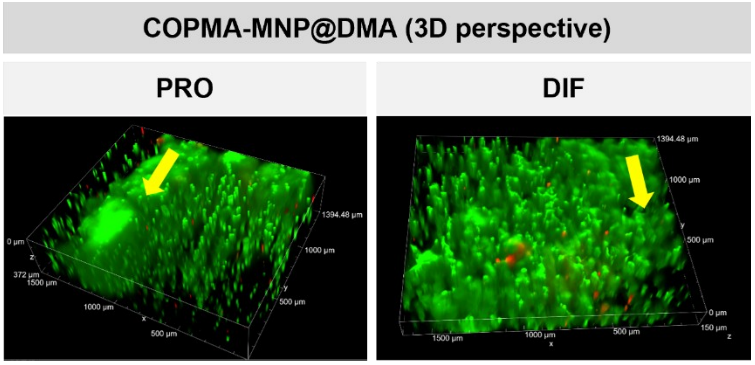

Figure S8. Live/dead staining images of COPMA-MNP@DMA constructs in 3D perspective on day 28. Yellow arrows indicate the magnetic field direction.

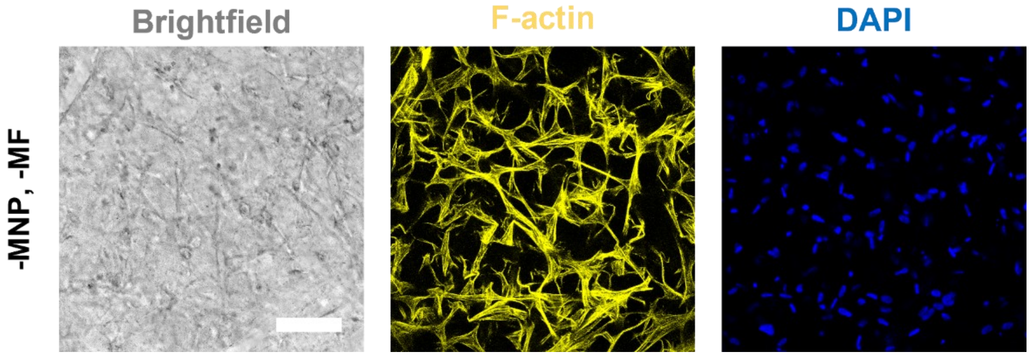

Figure S9. Confocal images of COPMA constructs without MNPs and MF, scale bar: 100 μm.

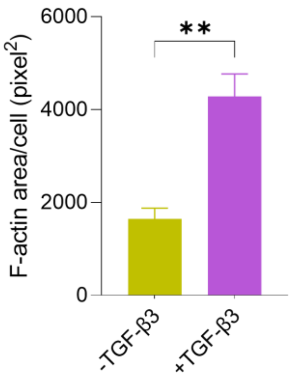

Figure S10. Comparison of F-actin area in isotropic COPMA-MNP@DMA constructs cultured with/without TGF-β3 (n ≥ 3, ***p* < 0.01).

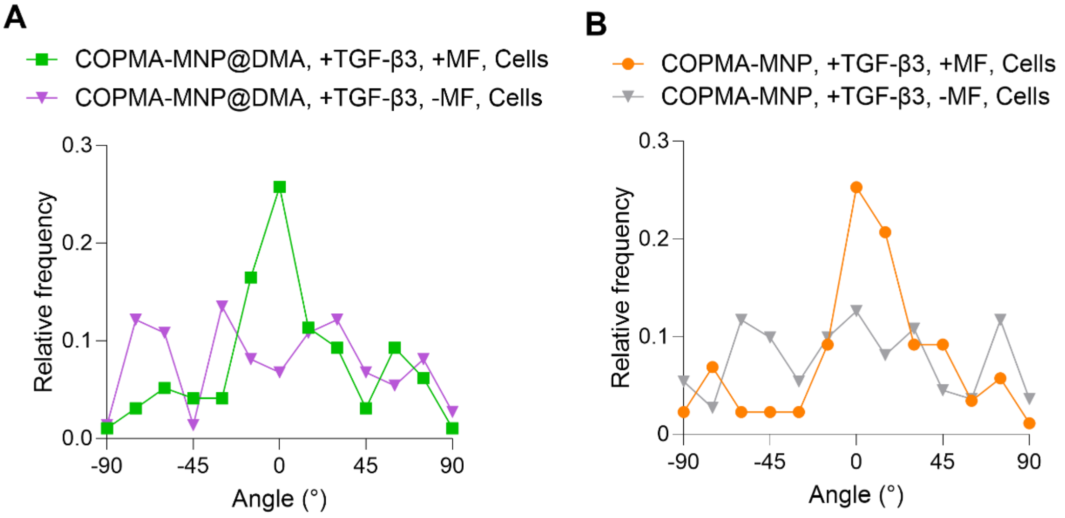

Figure S11. Angle distribution of cells in nanocomposite constructs with biochemical cues.

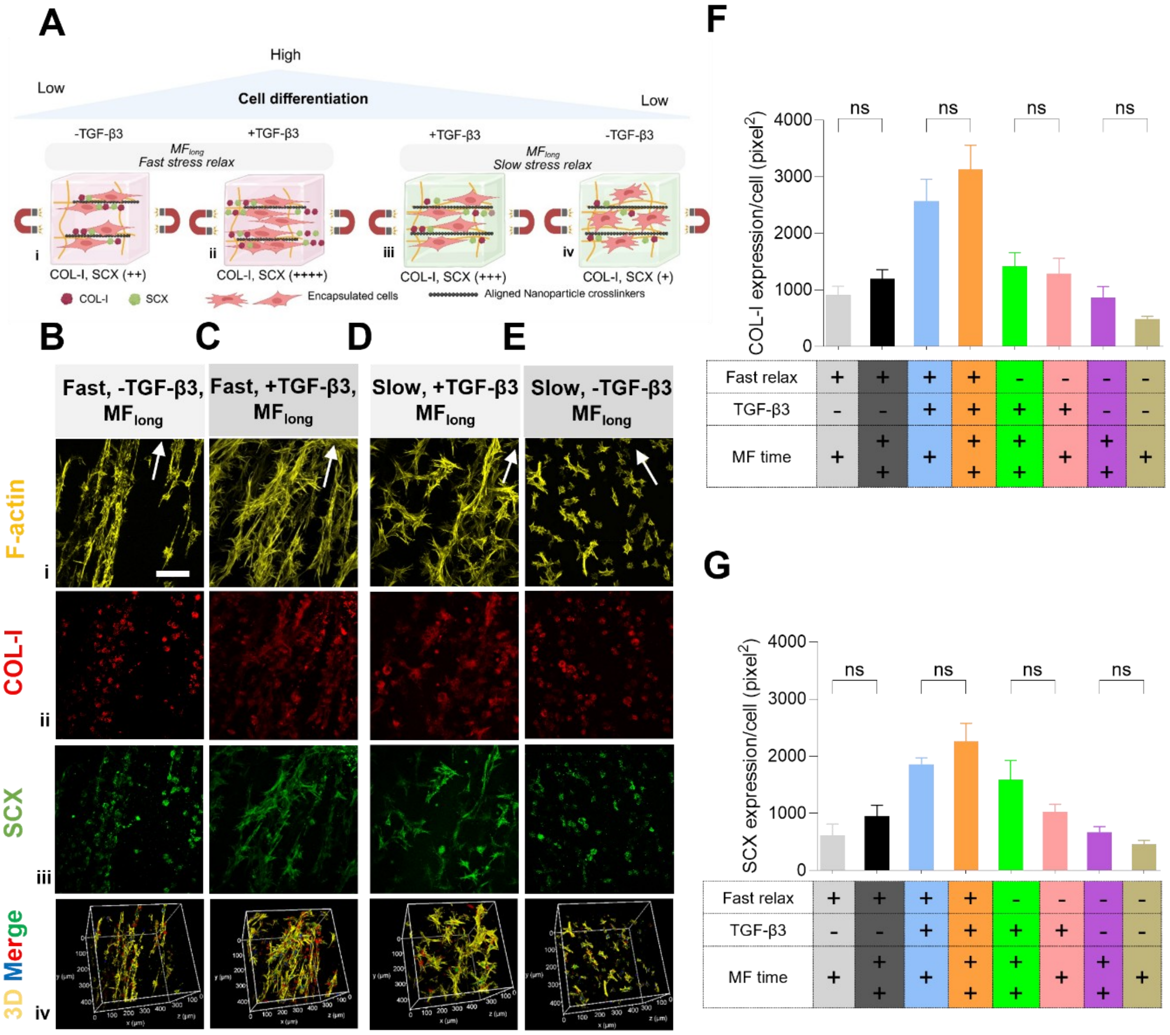

Figure S12. Comparison of effect of MF application time and biochemical cues on cell differentiation. A) Scheme of constructs on day 28. B-E) Immunostaining images of anisotropic constructs that were cultured with continuous magnetic field (MF_{long}) under proliferation (PRO) and differentiation (DIF) condition. Scale bar: 100 µm. White arrows indicate the direction of magnetic field. F-G) COL-I and SCX expressions in anisotropic constructs with magnetic field for 28 days (MF_{long}) and only for 3 min during gelation (MF_{short}) to compare the effect of continuous magnetic fields during cell culture on cell differentiation ($n \geq 3$, ns indicates no significant difference). Under proliferation conditions, COL-I and SCX expressions were similar in the same type of anisotropic constructs with MF_{long} (Figure S12B and S12E-G) and MF_{short} (Figure 6D and 6F), indicating the high efficiency of remote-control strategy. Under differentiation conditions, anisotropic constructs with continuous magnetic field exhibited slightly higher COL-I and SCX expression (Figure S12C-D, S12F-G), but no significant difference compared to those with only 3 minutes of magnetic field during gelation (Figure 6G-H). These findings indicated that continuous magnetic field during cell culture did not further promote hMSCs differentiation in the anisotropic constructs.

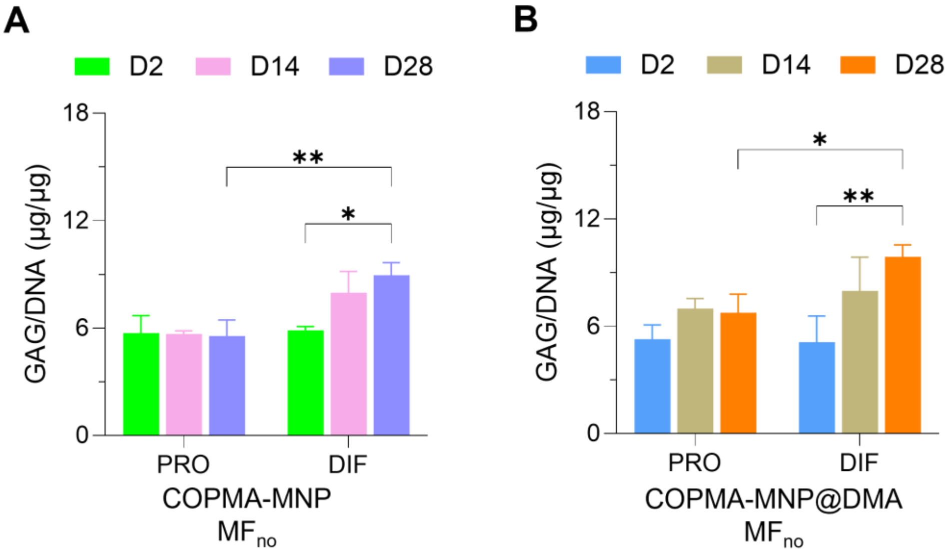

Figure S13. A) Normalized GAG production in isotropic COPMA-MNP constructs. B) Normalized GAG production in isotropic COPMA-MNP@DMA constructs ($n \geq 3$, *$p < 0.05$, **$p < 0.01$).
